# Complementary Surface Motifs Enhance NO_3_RR Performance in NiFe Alloys

**DOI:** 10.1002/cssc.202502337

**Published:** 2026-01-15

**Authors:** Jorin Dawidowicz, O. Quinn Carvalho, Shinnosuke Kamohara, Mohammad A. Zaki, Líney Árnadóttir, Kelsey A. Stoerzinger

**Affiliations:** ^1^ School of Chemical, Biological, and Environmental Engineering Oregon State University Corvallis Oregon USA; ^2^ Department of Chemical Engineering and Materials Science University of Minnesota Minneapolis Minnesota USA; ^3^ Physical and Computational Sciences Directorate Pacific Northwest National Laboratory Richland Washington USA

**Keywords:** adsorption, alloys, density functional calculations, electrocatalysis, nitrate reduction

## Abstract

Elemental first row transition metal electrocatalysts typically exhibit a tradeoff between Faradaic efficiency (FE) for the nitrate reduction reaction (NO_3_RR) and selectivity toward NH_4_^+^. Here, we find that NiFe alloys have high NO_3_RR FE *and* substantially higher NH_4_^+^ selectivity than Ni or Fe. We introduce “relative nitrate adsorption,” a simple descriptor of the difference in NO_3_* and H* binding strength that rationalizes experimental trends in reaction rate order. This descriptor is consistent with competitive adsorption demonstrated in a microkinetic model that shows Fe inclusion promotes NO_3_* adsorption and increased NO_3_RR FE, but cannot describe the higher NH_4_^+^ selectivity observed for NiFe alloys. In fact, calculated activation energies of subsequent reduction steps illustrate that no one active site motif can explain both improved FE and NH_4_^+^ selectivity. Instead, our experimental and computational findings indicate NO_2_* deoxygenation is promoted by Ni‐rich active sites, whereas NO* dissociation is promoted by both surface Fe atoms and an underlying Fe lattice. These findings suggest that NiFe alloys leverage local site diversity via a spillover mechanism, explaining why the performance enhancements are similar regardless of the specific Ni/Fe ratio.

## Graphical abstract

Mixed NiFe surface sites promote critical nitrate reduction reaction steps relative to pure Ni(111) and Fe(110), leveraging multiple adjacent sites to enhance ammonium selectivity and overall Faradaic efficiency.

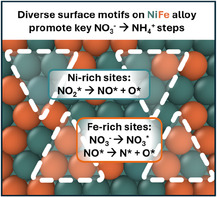

## 1 Introduction

The electrochemical nitrate reduction reaction (NO_3_RR) provides a promising approach for converting waste nitrate into dinitrogen gas or value‐added chemicals [1, 2]. Targeting ammonium as the product, NO_3_RR benefits include removing toxic nitrate from water and reducing reliance on energy‐intensive Haber‐Bosch ammonia synthesis. However, NO_3_RR follows a complex reaction network producing many intermediately reduced products and typically occurs at electrochemical potentials where the hydrogen evolution reaction (HER) competes for both protons and electrons (reducing Faradaic efficiency, FE) [2, 3, 4, 5, 6].

Among elemental first‐row transition metals, a general inverse scaling between selectivity toward NH_4_^+^ (with NO_2_^−^ as the dominant secondary product) and overall NO_3_RR FE limits catalyst design [4]. This tradeoff has been attributed to metals with a low d‐band center being limited by NO bond cleavage, and metals with higher d‐band centers being challenged by NO_2_^−^ reduction [7]. Co demonstrates a near‐optimal balance between NO_3_RR FE and NH_4_^+^ selectivity [8, 9, 10], but is toxic and not abundantly sourced [11, 12]. Alloying has emerged as a strategy to tune electrochemical performance while leveraging more abundant and less toxic elements [13]. However, strategies to select alloying elements, and the mechanistic route by which given elemental pairs improve selectivity, remain unclear.

In alloy catalysts, performance improvements have been attributed to global modification of electronic structure (e.g. shifts in d‐band center) [4, 14, 15, 16], lattice‐mediated geometric or strain effects [17, 18, 19], or a more localized picture where distinct site types promote different reaction steps, sometimes referred to as a dual active‐site or bifunctional catalyst [20, 21, 22, 23]. For a dual‐site mechanism where one element promotes adsorption of, e.g., H* and another NO_3_* [20], or one promotes NO_3_^−^ to NO_2_^−^ and another NO_2_^−^ to NH_3_ [23], rates and product distributions should have distinct composition dependence. However, most studies to date focus on a single bulk composition or nanostructures whose inherent roughness could introduce mass transport considerations, which can obscure kinetic insights for systems with soluble intermediates, like NO_2_^−^ [24, 25].

Here, we alloy nickel and iron—selected because their d‐band centers fall on either side of cobalt's, but are different enough to likely result in different rate limiting steps for NO_3_RR [4]. We perform electrochemical measurements on NiFe alloy foils, enabling direct assessment of how local surface composition governs NO_3_RR performance without confounding effects from nanoparticle morphology. These alloys achieve significantly greater NH_4_^+^ selectivity than either pure metal, with NO_3_RR FE similar to pure Fe and higher than Ni. Importantly, these metrics are similar across alloys ranging from 20 to 65 at% Fe. We use density functional theory (DFT) calculations of key NO_3_RR intermediates on NiFe surface alloy slabs to compare adsorption and activation energies for all possible adsorption site motifs, i.e., the distinct combinations of Ni and Fe atoms forming a threefold hollow site, and to quantify how much the surface–adsorbate interaction is governed by motif composition vs. the surrounding lattice. We demonstrate that the relative nitrate adsorption energy (Δ*E*_ads,H__‐__NO3_) scales with local site composition and rationalizes trends in NO_3_RR rate order consistent with microkinetic modeling. Even so, the experimental voltage dependence for alloys indicates that competitive adsorption is concurrent on multiple types of mixed NiFe sites. Computational analysis of reaction energetics further reveals that no one site configuration alone can explain both increased NO_3_RR FE and NH_4_^+^ selectivity. We find Ni‐rich sites have lower barriers for NO_2_* deoxygenation, whereas Fe‐rich sites and the surrounding Fe lattice both facilitate NO* dissociation, consistent with our spectroscopic investigations. Importantly, the multiatomic configuration of the active site necessitates explicit calculation of activation barriers due to deviations from BEP relations. The preference of NiFe alloys to exhibit dispersed, mixed surface sites (see Figure S3 and Table S3) and independence of product distribution on composition suggest that these alloys utilize multiple adjacent bridging and three‐fold hollow active sites via a spillover mechanism to achieve high NO_3_RR FE and NH_4_^+^ selectivity.

## 2 Results and Discussion

**FIGURE 1:**
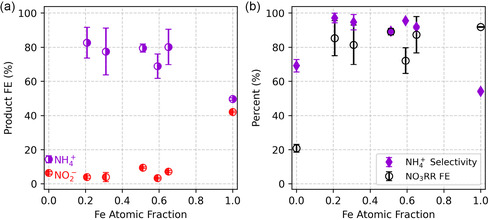
(a) Faradaic efficiency for NH_4_^+^ and NO_2_^−^ produced via NO_3_RR using Ni wire, Fe foil, and Ni–Fe alloy wire cathodes in 100 mM pH 7 sodium phosphate buffer with 100 mM NaNO_3_ at −0.5 V vs RHE after passing 0.097 e^−^/NO_3_^−^. Data values are averaged from three measurements each. Error bars are ±1 standard deviation. (b) Overall FE for NO_3_RR (the sum of the FEs in (a)) and electron selectivity toward NH_4_^+^.

The product distribution for NO_3_RR on a series of NiFe alloy cathodes was quantified in neutral media at −0.5 V versus the reversible hydrogen electrode (RHE) (Figure 1). Overall NO_3_RR FE is higher on Fe than Ni, while NH_4_^+^ selectivity is higher on Ni, consistent with previous reports (Table S1) [4].

NiFe alloys show much higher NH_4_^+^ selectivity than either Ni or Fe, with NO_3_RR FE comparable to Fe. For example, Ni_0.79_Fe_0.21_ shows a dramatic increase in NH_4_^+^ FE from 14% for pure Ni to over 80% for the alloy, while maintaining a low NO_2_^−^ FE (<5%). The improved NO_3_RR FE of the alloys compared to pure Ni suggests that the presence of Fe promotes NO_3_RR over HER, likely by enhancing NO_3_^−^ adsorption or its subsequent reduction to NO_2_*, which are often presumed to be the rate‐determining steps [26, 27]. The improved NH_4_^+^ selectivity of the alloys relative to pure Fe indicates that the presence of Ni stabilizes the NO_2_* intermediate against desorption, and/or facilitates its further reduction.

To better understand these distinctions in product distribution, we leverage reaction rate order, defined as the logarithmic dependence of current passed on the concentration of nitrate in solution. A maximum in rate order occurs because increasingly cathodic potentials increase both the rate of NO_3_RR and the coverage of H*, where the latter blocks sites for NO_3_* adsorption. The potential of maximum rate order (*V*_max_ _rate_ _order_) is less cathodic on Ni than Fe, indicating weaker binding of NO_3_* on Ni that allows H* to compete for sites more readily [4].

**FIGURE 2:**
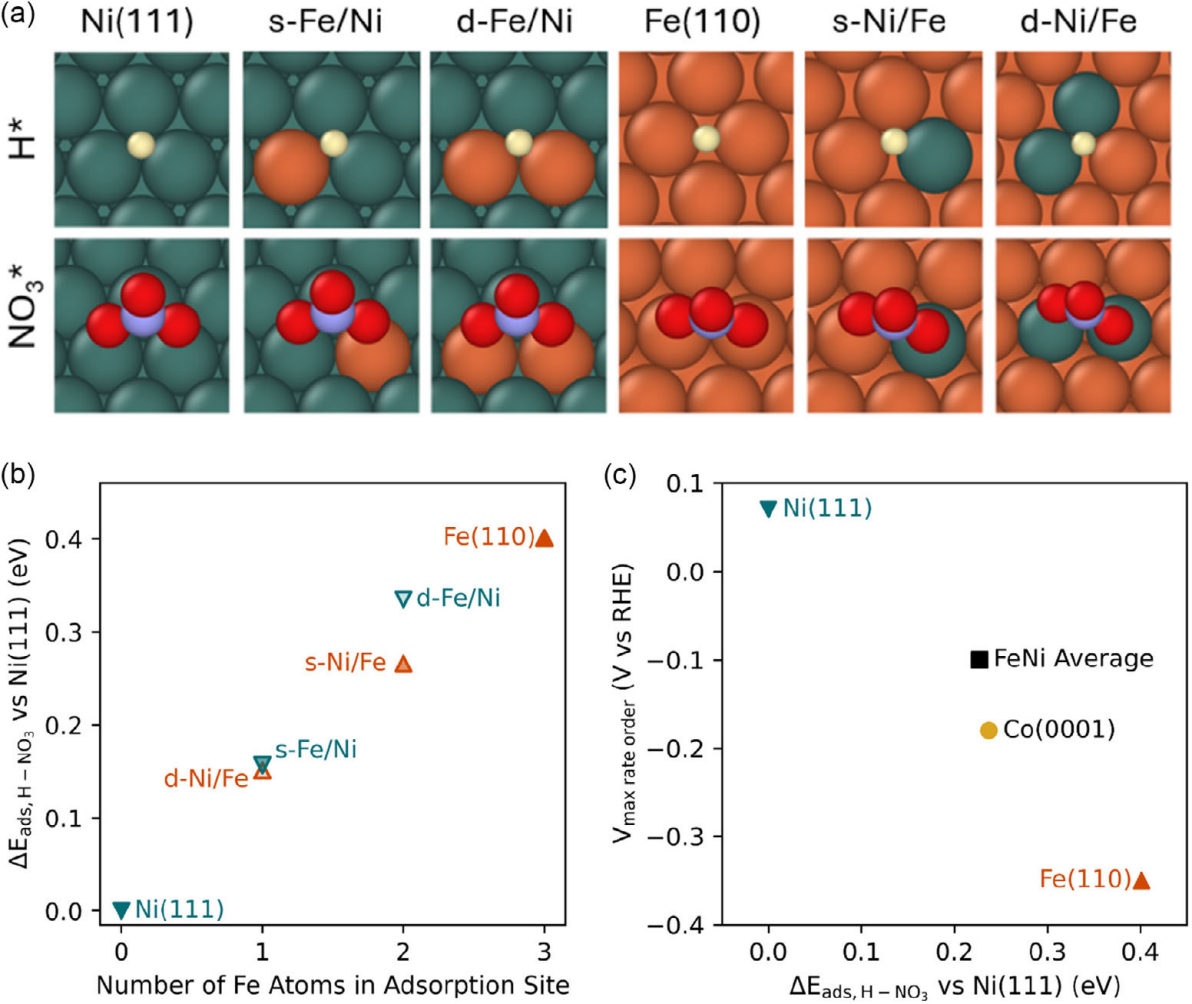
(a) Lowest energy adsorption configurations for H^*^ (top row) and NO_3_* (bottom row) on NiFe surface alloys (atom colors: Ni—teal; Fe—orange; H—off white; O—red; N—lavender). (b) Δ*E*_ads,H__‐__NO3_, for the different motifs. (c) Electrochemical potentials of maximum NO_3_RR rate order (*V*_max rate order_) for Ni, Co, and Fe (experimental data from Carvalho et al*.*, JACS 2022) [4], and for Ni_0.49_Fe_0.51_ (measured in this work), plotted against Δ*E*_ads,H‐_ _NO3_. The “FeNi average” point uses the experimentally measured *V*_max rate order_ for Ni_0.49_Fe_0.51_ and the average Δ*E*_ads,H__‐__NO3_ across all mixed hollow sites from panel (b).

To disentangle the influence of surface and bulk composition on competitive adsorption, we calculate H* and NO_3_* adsorption energies on Ni(111), Fe(110), and mixed threefold hollow surface motifs (2Fe1Ni and 2Ni1Fe). We model Ni(111) and Fe(110) as these represent the most closely packed and stable facets of face‐centered cubic (fcc) Ni and body‐centered cubic (bcc) Fe, respectively. The mixed motifs are designated (Figure 2a) as single (s‐) or double (d‐) substitutions of Ni on Fe(110) or Fe on Ni(111). Angle‐resolved XPS measurements (Figures S9 and S10) after electrochemical cycling reveal that the surface composition of the alloys trend with that of bulk composition, but may be slightly enriched with Fe. This characterization, considered alongside substitution energy calculations (Table S3), indicates that mixed Ni–Fe motifs are likely present on all NiFe alloy surfaces considered here. We note that experiments here were constrained to electrochemical potentials where bulk Pourbaix diagrams predict Ni and Fe in metallic form.

Consistent with conclusions drawn from rate order analysis, NO_3_* adsorption is energetically favored over H* adsorption to a greater degree on Fe(110) than on Ni(111) (Table S3). We quantify this via a “relative nitrate adsorption” descriptor (Δ*E*_ads,H‐_ _NO3_), defined as the energy difference between NO_3_* and H* adsorption at a given surface motif referenced to a fcc hollow site on Ni(111) (Figure 2b; full equations and discussion of the influence of including implicit solvation in Figure S4 are provided in the Supporting Information). Positive values indicate that a motif binds NO_3_* more strongly (or H* more weakly) than Ni(111). For Ni(111), Fe(110), and Co(0001), there is a clear linear trend in which increasing Δ*E*_ads,H‐_ _NO3_ correlates with more cathodic *V*_max_ _rate_ _order_ (Figure 2c), validating both metrics as indicators of competitive adsorption behavior.

Across the range of motifs, the computed Δ*E*_ads,H__‐__NO3_ increases linearly with Fe content (Figure 2b) and site composition is a stronger determinant than host lattice identity. For example, threefold hollow sites containing two Ni atoms and one Fe atom have Δ*E*_ads,H__‐__NO3_ about 0.15 eV greater than Ni(111), regardless of host lattice.

Experimentally, the reaction rate order for Ni_0.49_Fe_0.51_ exhibits a broad maximum around −0.1 V vs. RHE and drops off at more cathodic potentials (Figure S5). This contrasts with pure Fe, which has a maximum in rate order at more cathodic potentials, as well as a more negative onset for NO_3_RR (Figure S6). Compared to Ni, Ni_0.49_Fe_0.51_ has a more cathodic *V*_max rate order_, indicating that Ni_0.49_Fe_0.51_ binds NO_3_* more strongly (H binding is relatively insensitive to composition and host, Figure S11), in agreement with gas‐phase adsorption quantified with ambient pressure XPS (Figure S8). The broad shape of *V*_max_ _rate_ _order_ on the alloy resembles a superposition of the rate order vs. potential curves predicted by the microkinetic model for each individual motif (Figure S5). Physically, the broadness of *V*_max_ _rate_ _order_ on the alloy indicates a wider potential window in which NO_3_* and H* adsorption are thermodynamically competitive, likely due to competitive adsorption occurring concurrently on different surface motifs. Considering the measured rate order as arising from a superposition of the behavior from the different active site motifs modeled here provides a picture where NO_3_* is sequentially displaced from sites with increasing Fe content at more reducing potentials. A similar conclusion can be drawn from plotting *V*_max_ _rate order_ for Ni_0.49_Fe_0.51_ against the average Δ*E*_ads,H__‐__NO3_ calculated for all mixed motif/host lattice combinations, approximating the distribution expected in the equimolar alloy. The resulting “FeNi Average” data point fits reasonably well into the linear pure‐metal trend, indicating that adsorption at the alloy surface can be modeled by averaging the adsorption behavior at the surface's constituent motifs.

Similar to the NiFe system, previous studies report that adding Cu to Ni lead to an increase in relative NO_3_* adsorption [4], evidenced by a cathodic shift in the potential of maximum rate order. However, the improved NO_3_RR FE of NiCu alloys comes at the *expense* of NH_4_^+^ selectivity, whereas alloying Fe with Ni favors NO_3_RR while *maintaining* high NH_4_^+^ selectivity. This distinction may arise from Fe having a higher d‐band center than Ni [7]. Our density of states (DOS) calculations show that the d‐band centers of surface atoms are influenced by both host lattice and local site composition. NO_3_* adsorption energies correlate linearly with the average d‐band center of the three atoms that make up the motif (Figure S12), whereas N* and O* adsorption are more strongly affected by host lattice identity (Figure S11).

**FIGURE 3:**
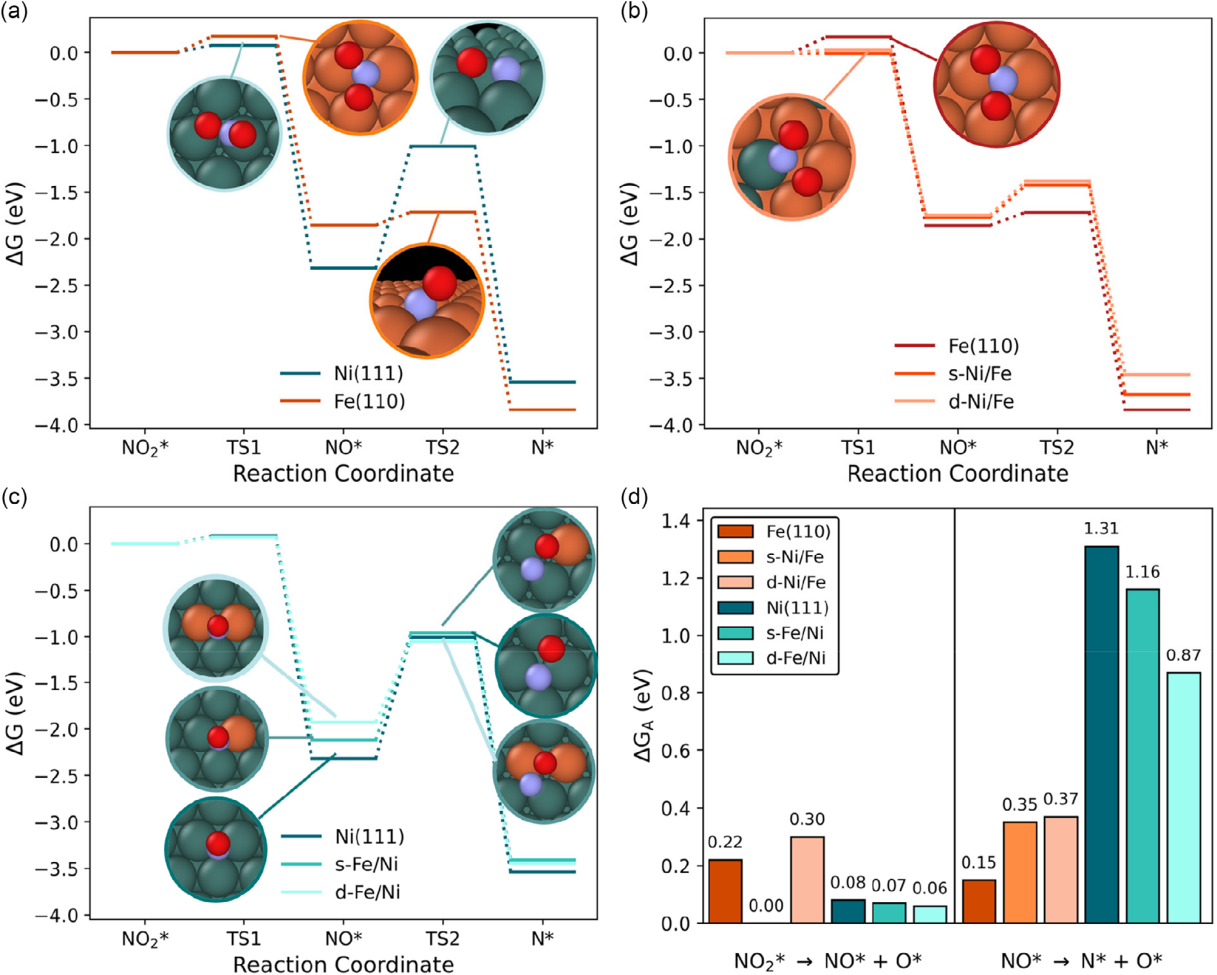
Free energy diagrams for NO_2_* deoxygenation and NO* dissociation steps on (a) Ni(111) and Fe(110); (b) Ni(111) with 0, 1 (s‐Fe/Ni), and 2 (d‐Fe/Ni) Fe atoms substituted in the surface; and (c) Fe(110) with 0, 1 (s‐Ni/Fe), and 2 (d‐Ni/Fe) Ni atoms substituted in the surface. (d) Summary of activation energy barriers (ΔG_A_) for NO_2_* deoxygenation and NO* dissociation on each surface.

To further investigate how NiFe alloys promote NH_4_^+^ selectivity, we calculated activation barriers for two important mechanistic steps for the reduction of NO_3_* beyond NO_2_* (Figure 3): NO_2_* deoxygenation (NO_2_* + * → NO* + O*) and NO* bond breaking (NO* + * → N* + O*). Although NO bond breaking of a *NOH intermediate is not considered explicitly here, scaling relations would suggest a similar trend across chemistries. We note that in the electrochemical environment these processes could occur via proton coupled electron transfer to yield water as a byproduct instead of adsorbed O [28], but that these calculations represent the effect of the active site composition on the bond breaking event.

For the Ni(111) and Fe(110) slabs, the calculated activation barriers (Figure 3a) are consistent with experimentally observed product distributions. The activation barrier for NO_2_* deoxygenation is low (0.08 eV) on pure Ni(111) and higher (0.21 eV) on Fe(110). This finding, together with the appreciable detection of NO_2_^–^ in solution for Fe cathodes (but not alloys or Ni), suggests the barrier for NO_2_* deoxygenation is greater than that of its dissolution on Fe(110). Conversely, the NO* dissociation barrier and reaction energy are both much higher on Ni than on Fe, consistent with our previous finding that Fe promotes NO* dissociation more than Ni [7]. We find stabilization of the N* + O* final state is much stronger on Fe(110) than on Ni(111). The transition state on Fe(110) is a tilted NO* arrangement that is closer to the initial state, whereas on Ni(111) the transition state occurs after N–O bond breakage.

Incorporating one or two Fe atoms into a Ni(111) surface impacts the activation barriers of NO_2_* and NO* dissociation to different extents (Figure 3b,d). The first step, NO_2_* deoxygenation to NO* + O*, is largely insensitive to surface composition, with low activation barriers across all Ni(111)‐hosted surfaces. The low barriers and favorable reaction energies support facile NO_2_* conversion and align with the low NO_2_^−^ FE observed experimentally for both pure Ni and NiFe alloys. In contrast, the barrier for NO* dissociation is more strongly affected by surface composition. Adding Fe into the Ni(111) surface lowers the activation barrier, primarily via destabilization of the initial NO* state. However, the barrier remains notably higher than for the same mixed motifs on Fe(110), demonstrating the influence of the host lattice.

Ni incorporation in the Fe(110) surface effectively eliminates the NO_2_* dissociation activation barrier (Figure 3c,d), as the NO* intermediate is stabilized by a Ni‐Fe bridge following deoxygenation. The enhanced NO_2_* dissociation pathway could enable rapid conversion of NO_2_* into downstream products, consistent with the greatly reduced FE for NO_2_^−^ observed on NiFe alloys compared to pure Fe. This improved efficiency comes at the expense of the NO* dissociation barrier, which increases ∼0.2 eV in the presence of Ni.

We note that these alloyed surfaces uniquely impact activation energetics in comparison to thermodynamic reaction energetics. Commonly assumed Brønsted–Evans–Polanyi (BEP) scaling relations [29] hold for NO* dissociation, but not for NO_2_* deoxygenation (Figure S13), due to different bi‐ and tridentate adsorption configurations of NO_2_* on Ni(111)‐hosted and Fe(110)‐hosted motifs (Table S3), which result in different transition state geometries (similar to those pictured in Figure 3a) [30].

Together, the differences in these activation barriers show that alloying redistributes activity across distinct motifs: Ni‐rich environments promote NO_2_* deoxygenation, while Fe‐rich environments facilitate NO* dissociation. Yet high NH_4_^+^ selectivity requires both steps to proceed rapidly, and no single surface motif minimizes both barriers. Therefore, the sharp increase in ammonia selectivity observed for alloys is best explained by a multisite mechanism, in which the lowest energy paths require different surface motifs. In principle, this explanation is similar to the tandem mechanism proposed by Majhi et al. [23], who recently achieved high NO_3_RR FE and NH_4_^+^ selectivity using NiFe alloy nanoflowers, and used adsorption energy calculations to argue that NO_3_* binds and is reduced to NO_2_* on Fe sites, but that further reduction steps occur on Ni. However, our activation barrier calculations show that while Ni‐rich sites promote NO_2_* reduction, they also impart a strong kinetic penalty on NO* dissociation, indicating that the NO* likely migrates back to Fe‐rich sites, where it is both most strongly bound and has the smallest activation barrier for bond cleavage. As noted above, this utilization of multiple active sites also explains the broad window of *V*_max rate order_ for Ni_0.49_Fe_0.51_ compared to Ni or Fe, which illustrates differences in competitive adsorption of NO_3_* and H* across diverse NiFe site motifs present on alloy surfaces.

## 3 Conclusions

Across a wide range of compositions, NiFe alloys outperform their parent metals in NO_3_RR, achieving both high NH_4_^+^ selectivity and high overall FE. The difference between NO_3_* and H* adsorption energies scales with local site composition and captures differences in the experimental potential of maximum rate order, rationalizing improvements in NO_3_RR FE by suppressing HER. Activation barriers for individual reaction steps calculated with DFT show Ni‐rich motifs lower activation barriers for NO_2_* deoxygenation, whereas Fe‐rich motifs and the Fe(110) lattice strongly promote NO_3_* adsorption and NO* dissociation. These findings suggest that the superior performance of NiFe alloys occurs because intermediate species have access to a variety of complimentary surface sites over the course of the reaction. More broadly, the results demonstrate how disordered alloy surfaces can combine favorable attributes of distinct local motifs and host environments through a spillover mechanism, and highlight surface composition and host lattice as useful and distinct levers for tuning catalytic activity in alloys.

## Supporting Information

Additional supporting information can be found online in the Supporting Information section. The authors have cited additional references within the Supporting Information.[31, 32, 33, 34, 35, 36, 37, 38, 39, 40, 41, 42, 43, 44, 45, 46, 47, 48, 49, 50, 51, 52] **Supporting** **Fig.** **S1:** Characterization of as received samples of (a) Ni wire, (b) Ni0.68Fe0.31 wire, (c) Ni0.49Fe0.51 wire, (d) Ni0.42Fe0.49 wire, and (e) Fe foil. Left: SEM micrograph, center: Ni Lα1,2 EDS map, right: Fe Lα1,2 EDS map. For Ni and Fe, the EDS spectrum is shown in lieu of a map of the omitted element. **Supporting Fig. S2** **:** X‐ray diffraction (XRD) θ‐2θ scan, λ_Cu_ = 1.54 Å with generator set to 40 kV and 45 mA. **Supporting Fig. S3** **:** Relative abundance of 3Ni, 1Fe2Ni, 2Fe1Ni, and 3Fe hollow sites on a simulated close‐packed hexagonal surface consisting of randomly distributed Ni and Fe atoms, as a function of overall surface composition. Decreasing the concentration of the majority element to below approximately 0.8 results in mixed motifs outnumbering pure majority‐element motifs. **Supporting Fig. S4** **:** Comparisons of a) H* and b) NO_3_* adsorption energies calculated with and without implicit solvation. **Supporting Fig. S5** **:** a) Experimentally determined NO3RR rate order with respect to NO_3_^−^ concentration across a range of potentials for Ni_0.5_Fe_0.5_ (measured here), Ni, and Fe (adapted from Carvalho et. al1 with permission. Copyright 2022 American Chemical Society); b) logarithmic graph of steady state current density vs nitrate concentration measured for Ni_0.5_Fe_0.5_ at five representative potentials – slope of linear fit used to determine rate orders in panel a; c) Modeled NO_3_RR rate order (with respect to NO_3_^–^ concentration) versus electrochemical potential, using parameters listed in **Table S4**; (d) Relation between the modeled potential of maximum rate order, E_max rate_ order, and the relative nitrate adsorption energy, ΔG_H*‐NO3__*_. **Supporting Fig. S6** **:** Representative CV sweeps (unstirred) for HER only (blue) and with nitrate (red) of a) Ni wire, b) Ni_0.49_Fe_0.51_ wire, c) Ni_0.41_Fe_0.59_ wire, and d) Fe foil in 100 mM pH 7 sodium phosphate buffer with and without 10 mM of sodium nitrate, with a scan rate of 10 mV/s. The mass transfer limited feature around ‐0.4 V vs RHE arises from phosphate deprotonation.1 Current densities are normalized by electrochemical surface area as determined from capacitance measurements (**Figure S7**). **Supporting Fig. S7** **:** Measurement of double layer capacitance for estimation of ECSA; the current at the potential indicated by the dashed line was used as a function of scan rate to extract the capacitance, accounting for any minor Faradaic baseline giving rise to asymmetry in the cathodic and anodic sweep. The surface area for normalization of CVs was estimated assuming a specific capacitance of 40 μF/cm^2^ 20a) Ni, b) Ni_0.49_Fe_0.51_, c) Ni_0.41_Fe_0.59_, d) Fe. **Supporting Fig. S8** **:** a) Core level N 1s spectra (560 eV hν) for a series of electrodeposited Fe_x_Ni_1‐x_ alloy oxides after cleaning in ultrahigh vacuum (UHV) (light gray trace) and during exposure to 1 mTorr of gaseous NO (black trace). b) Ratio of N 1s and TM 3p intensities (with increasing Fe content: 0.23, 0.52, 2.07, 1.43, 0.27) measured during 1 mTorr NO exposure. **Supporting Fig. S9** **:** Ni and Fe 3p XPS spectra for Ni_0.8_Fe_0.2_ and Ni_0.36_Fe_0.64_ (wt%) foils following NO_3_RR measurements measured with normal and shallow (12o) emission. Units for intensity are counts per second (CPS), but in order to aid visual comparison, an offset is applied, and the shallow emission CPS are multiplied by 10 to counteract their weaker photoelectron signal. Although shallow emission (surface sensitive) measurements suggest an oxidized surface (in contrast to a reduced bulk), the ex situ nature of this measurement precludes conclusions regarding the active valence state during electrochemistry. **Supporting Fig. S10:** Estimated atomic iron fractions by XPS following NO_3_RR measurements of the foil surfaces (from shallow emission) and bulk (from normal emission), where the composition noted on the x‐axis and legend is wt%, corresponding to Ni_0.79_Fe_0.21_ and Ni_0.37_Fe_0.63_ atomic fraction based on the purchased bulk composition. **Supporting Fig. S11:** Differences in ΔE_ads_ for various adsorbates on equivalent a) hollow and b) bidentate adsorption sites, on different host lattices (Ni(111) or Fe(110)). All adsorbates bound more strongly (lower ΔE_ads_) to sites on the Fe(110) lattice. Values shown are calculated as ΔE_ads_ _, Ni(111) host_ ‐ ΔE_ads_ _, Fe(110) host_. **Supporting Fig. S12:** ΔE_ads_ of NO_3_ (a,b) and N (c,d) on each surface motif plotted against d‐band centers calculated for the entire surface slab (b,d) or averaged from the three surface motif atoms only (a,c). Data points are color coded based on the atom content of the surface motif (3 Fe – red, 2 Fe and 1 Ni – yellow, 2 Ni and 1 Fe – green, 3 Ni – blue). Pearson correlation coefficients (r) are show the linear correlation between these values. There are strong negative correlations (indicated by r values close to ‐1) between surface motif averaged d‐band center and ΔE_ads_ _,NO__3_ (r = ‐0.937) and between slab average d‐band center and ΔE_ads,N_ (r = ‐0.929), reflecting the two adsorbates’ greater sensitivity to surface and bulk composition, respectively. **Supporting** **Fig.** **S13:** Activation free energy barrier vs free energy of reaction for (a) NO_2_* deoxygenation and (b) NO* dissociation steps on each surface motif. For NO* dissociation (b), there is a linear correlation between these values, in line with BEP scaling relations. However, for NO_2_* deoxygenation (a), a linear trend is not apparent, suggesting that BEP scaling should not be assumed. **Supporting** **Fig.** **S14:** Reaction free energy diagram for N* + H* → NH* on Ni(111), s‐Ni/Fe, Fe(111), and s‐Fe/Ni. **Supporting** **Table** **S1:** Catalyst performance in 100 mM pH 7 sodium phosphate buffer with 100 mM NaNO_3_ at ‐0.5 V vs RHE (85% iR correction applied). **Supporting** **Table** **S2:** EDS composition. **Supporting** **Table** **S3:** Adsorption energies (E_ads_) in eV and preferred sites for various adsorbates. All adsorption energies were calculated using equation 4. Abbreviations indicate the preferred adsorption site: face‐centered cubic hollow (fcc), long bridge (lb), hollow, bidentate (b; dentate atoms indicated), and tridentate (t; dentate atoms indicated). Only configurations in which the adsorbates are directly bonded to each dopant atom are reported. **Supporting** **Table** **S4:** Surface Slabs and Substitution Energies (E_sub_) for Fe in Ni(111) and Ni in Fe(111). Esub values were calculated using equation 5. **Supporting** **Table** **S5:** Parameters used to produce microkinetic model results in **Figure S4**.

## Conflicts of Interest

The authors declare no conflicts of interest.

## Supporting information

Supplementary Material

## Data Availability

The data that support the findings of this study are available from the corresponding author upon reasonable request.
