# Supplementary material for: Complementary Surface Motifs Enhance NO3RR Performance in NiFe Alloys
Source: ChemSusChem. 2026 Jan 15;19(1):e202502337. doi: 10.1002/cssc.202502337 (PMC12808557; doi:10.1002/cssc.202502337)
Supplement: Supplementary file 1 — Supplementary Material [file CSSC-19-e202502337-s001.pdf]

Supplemental Information for: Complementary Surface Motifs Enhance NO3RR Performance in NiFe Alloys

Jorin Dawidowicz,[1] O. Quinn Carvalho,[1,†] Shinnosuke Kamohara,[2] Mohammad A. Zaki,[2] Líney Árnadóttir,[1,3,*] Kelsey A. Stoerzinger[1,2,‡,*]

[1] School of Chemical, Biological, and Environmental Engineering, Oregon State University, Corvallis, OR, 97331, USA

[2] Department of Chemical Engineering and Materials Science, University of Minnesota, Minneapolis, MN 55455

[3] Physical and Computational Sciences Directorate, Pacific Northwest National Laboratory, 902 Battelle Blvd. Richland, WA 99354, USA

** Corresponding email: Liney.Arnadottir@oregonstate.edu; zinger@umn.edu*

† Current address: National Renewable Energy Laboratory, Golden, Colorado 80401, USA

‡ Current address: Department of Chemical Engineering and Materials Science, University of Minnesota, Minneapolis, MN 55455

Contents

Methods 2

Experimental 2

DFT 8

Discussion of mixed site modeling: 11

Relative Nitrate Adsorption definition: 14

Assessing possible solvation effects: 14

NO_3RR rate order with respect to NO_3^- concentration 15

APXPS shows NiFe oxides adsorb more NO_x than FeO_x or NiO_x 20

Surface composition – Angle Resolved XPS 21

Sensitivity of adsorbates to host vs site composition 24

Shift in d-band energy from host lattice vs surface alloy motif 25

NO_2* Deoxygenation and NO* Dissociation BEP Scaling 26

N* Hydrogenation Energy 26

References 27

Methods

Experimental

Materials and general methods

The cathodes used in the electrochemical processes were high purity (≥99.9%) Ni wire and NiFe alloy wires purchased from Goodfellow, as well as Fe foil (Beantown Chemical, ≥99.5%). In all cases, the radius of curvature was >50 microns, such that no appreciable difference is expected in terms of diffusion of nitrate across the stagnant layer to the reactive surface for wire vs foil form factors. We observe comparable overall NO_3RR FE and NH_4^+ selectivity from a Ni wire to that of a Ni foil in our previous work,[1] further confirming this (**Table S1**).

Table S1. Catalyst performance in 100 mM pH 7 sodium phosphate buffer with 100 mM $NaNO_3$ at -0.5 V vs RHE (85% iR correction applied).

Catalyst form factor	Charge passed	NO3RR FE	NH4+ selectivity
0.1 mm diameter Ni wire, Goodfellow (present work)	0.097 e^-/NO_3^-	21 +/- 2%	69 +/- 4%
Ni foil, Goodfellow (reference 1)	0.2 e^-/NO_3^-	16 +/- 7%	70 +/- 8%

Alloy compositions used in order of increasing Fe weight percentage: 20%, 30%, 50%, 58%, and 64% (corresponding approximately to atomic ratios $Ni_{0.79}Fe_{0.21}$, $Ni_{0.69}Fe_{0.31}$, $Ni_{0.49}Fe_{0.51}$, $Ni_{0.41}Fe_{0.59}$, and $Ni_{0.35}Fe_{0.65}$). Characterization of representative samples via SEM (Thermo Fisher Scientific Apreo 2S), EDS (Oxford Instruments Ultimax 100 mm^2), and XRD (Rigaku SmartLab SE, λ_{Cu} = 1.54 Å) is provided in **Figure S1-S2**, and EDS compositional analysis agrees (**Table S2**) well with expected composition from the vendor. Measurement parameters were a 10 kV voltage, 6.4 nA current, ETD detector, and 10 mm working distance.

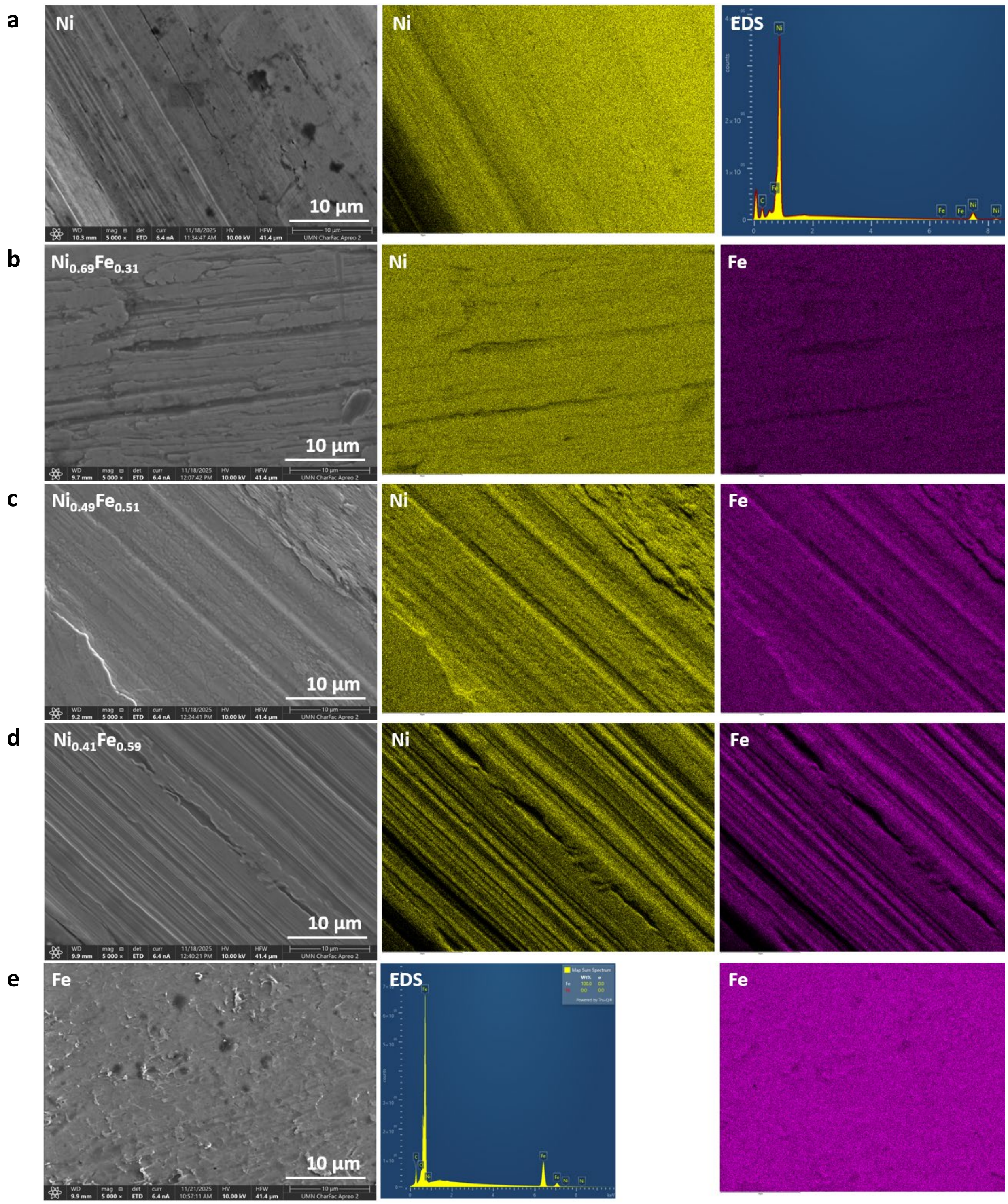

Figure S1. Characterization of as received samples of (a) Ni wire, (b) Ni0.68Fe0.31 wire, (c) Ni0.49Fe0.51 wire, (d) Ni0.42Fe0.49 wire, and (e) Fe foil. Left: SEM micrograph, center: Ni Lα1,2 EDS map, right: Fe Lα1,2 EDS map. For Ni and Fe, the EDS spectrum is shown in lieu of a map of the omitted element.

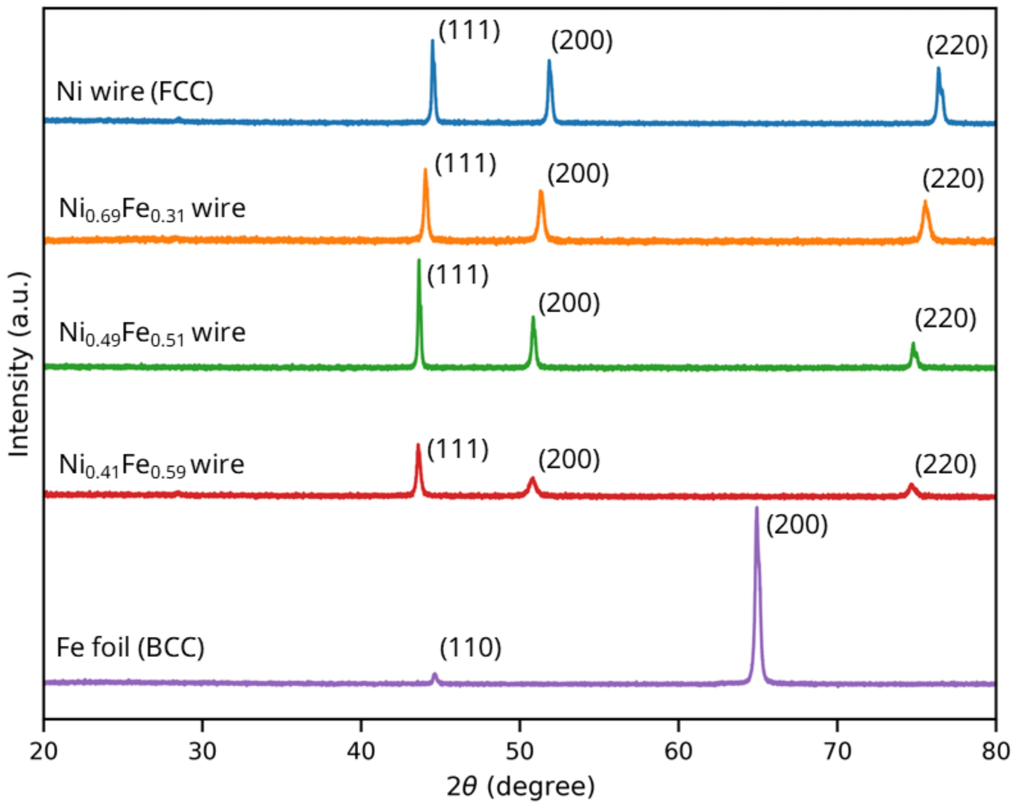

Figure S2. X-ray diffraction (XRD) θ-2θ scan, λ_{Cu} = 1.54 Å with generator set to 40 kV and 45 mA

Table S2. EDS composition

	EDS				Expected
	Fe at%	Ni at%	O at%	Fe/(Ni+Fe)	Fe/(Ni+Fe)
30 wt% Fe	23.5	71.8	4.7	24.7	31
50 wt% Fe	44.8	51.7	3.6	46.4	51
58 wt% Fe	51	43.2	5.8	54	59

Prior to electrochemical measurements, pure Ni and Fe samples were cleaned by etching in a 1:4 (v/v) ratio mixture of hydrochloric acid to millipure deionized water (DIW) for 30 seconds. Alloy samples were instead cleaned by abrasion with fine-grit sandpaper to avoid altering their surface composition through acid exposure.[2,3] Next, the samples were sonicated for 15 minutes in a vial of 18.2 MΩ water. These cleaning steps were followed by thorough rinsing with 18.2 MΩ water and drying with nitrogen gas.

The 100 mM phosphate buffer electrolyte (pH = 7) was prepared using Certified ACS Grade monobasic sodium phosphate (NaH_2PO_4) and dibasic sodium phosphate monohydrate (Na_2HPO_4 x H_2O) from Fischer Chemical in 18.2 MΩ water. The noted concentration of sodium nitrate was

achieved through addition of a stock solution ($NaNO_3$; EMD Millipore, ACS Grade). All electrochemical measurements were performed in UHP Ar-saturated electrolyte with a Biologic SP-300 potentiostat, controlled by EC-Lab software. Potentiostatic electrochemical impedance spectroscopy (PEIS) was run at open circuit to determine the solution resistance. This value was used to set up 85% manual iR compensation through EC-Lab for subsequent electrochemical steps. All experiments used a Ag/AgCl reference electrode experimentally calibrated to the RHE using a Pt wire and H_2 gas in the phosphate buffer electrolyte.

Rate order analysis

Cyclic voltammetry (CV) and chronoamperometry (CA) for rate order analysis was carried out in a three-electrode beaker cell filled with 70 mL electrolyte, using the NiFe sample as the working electrode with a Ni metal mesh connected to Ni wire as the counter electrode. Insulating epoxy resin was used to coat each cathode surface, leaving only a small (approximately 0.1 cm^2) surface area exposed. This surface area was quantified visually using ImageJ software.

The nitrate-free electrolyte was thoroughly stirred and sparged with argon gas for approximately thirty minutes, followed by initial CV and CA measurements. Sodium nitrate stock solution was then added to the cell via micropipette to achieve a series of desired nitrate concentrations. After each nitrate addition, the cell contents were mixed with a magnetic stir bar and sparged with Ar gas to displace any O_2 gas present. Then, at each concentration, steady state CA was used to hold the cell at a series of potentials in 40 mV increments for 30 seconds each, during which time the steady state current was measured.

Conversion/Chronoamperometry:

CA was carried out in glass H-cells, with two compartments separated by a Nafion 117 membrane. The anolyte contained 100 mM sodium phosphate buffer and a graphite rod counter-electrode. The catholyte contained a coiled NiFe alloy wire working electrode and secondary frit with an Ag/AgCl reference electrode in it, and was sparged by Ar gas. After preliminary CVs in a nitrate-free buffer, sodium nitrate stock solution was added to the catholyte to bring its total nitrate concentration up to 100 mM. After approximately five minutes of stirring and re-sparging, a 100 µL sample of the catholyte (“t_0”) was taken and added to 400 µL of 18.2 MΩ water. Then, PEIS at the open circuit potential was performed to correct the applied potential for cell resistance. CA was performed, passing a predetermined amount of charge, Q, through the cell at a constant applied potential of -0.5 V vs RHE. Q was chosen using Equation 1, below, to be the amount of charge needed to convert 4.83% of the nitrate present in the catholyte to nitrite, assuming 100% FE for that reaction.

$$Q = V * c_{NO_3} * n_{NO_2} * F \qquad (1)$$

Where Q is the charge passed through the cell, V is the volume of the catholyte, c_{NO3} is the concentration of nitrate anions in the catholyte, n_{NO2} is 2, the number electrons involved in nitrate-to-nitrite reduction, and F is the Faraday constant. This is equivalent to passing 0.097 mol e^-/mol NO_3^- initially in solution.

After the CA was complete, another 100 µL sample of catholyte ("t_1") was added to 400 µL of 18.2 MΩ water.

The t_0 and t_1 samples were stored in a refrigerator for no more than two weeks before being further analyzed using methods described in the next section.

Conversion Sample Analysis

After further diluting each sample by mixing 15 µL of sample with 1485 µL of 18.2 MΩ water, ion chromatography (IC; Thermo Scientific Dionex ICS-5000) was used to measure the concentrations of nitrate and nitrite. The IC was operated with a Dionex IonPac CG16 5 x 50 mm guard column, eluent flow rate of 0.7 mL/min 15 mM sodium hydroxide (NaOH; Thermo-Fisher), 40 °C column temperature, and 25 µL sample volume. Standard curve and elution time were determined from a series of sodium nitrite ($NaNO_2$), sodium nitrate ($NaNO_3$; EMD Millipore, ACS Grade), and sodium phosphate buffer aqueous solutions.

Ammonium concentrations in the samples were determined by UV-Vis spectrophotometry. 10 µL volumes of each sample were loaded into a 300 µL 96-well plate along with 40 µL of 18.2 MΩ water, 150 µL of trisodium citrate solution (2.5 g of trisodium citrate in 50 mL of 18.2 MΩ water), 50 µL of 2-phenylphenol (2PP) sodium salt solution (consisting of 3.0 mg sodium nitroprusside, 644 mg of 2PP, and 20 mL of 18.2 MΩ water), and 25 µL of buffered sodium hypochlorite solution (2.0 mL of Clorox (about 7.4%), 200 mg trisodium phosphate, and 20 mL 18.2 MΩ water).

The objective of these analyses was to determine the concentrations of nitrite, nitrate, and ammonium in order to calculate the overall NO_3RR Faradaic Efficiency (FE) and the selectivity of the reaction towards ammonium at the reaction conditions tested. The FE of a species i was calculated as:

$$FE_i(Q) = \frac{\left(c_i(Q) - c_i(0)\right) n_i F}{\frac{Q}{V}} \times 100\% \qquad (2)$$

where $c_i(Q)$ is the concentration of species *i* in the t_1 sample, which had an amount of charge Q passed through it, $c_i(0)$ is the concentration of species *i* measured in the t_0 sample (sampled before any charge was passed), n_i is the number of electrons used in converting one molecule of nitrate to one molecule of species i (n_{NO2^-} = 2; n_{NH4+} = 8), F is the Faraday Constant (96,485 C/mol e^-), Q is the charge passed, and V is the volume of electrolyte in the cathode cell.

The selectivity of each species, S_i, was calculated as

$$S_i(Q) = \frac{FE_i(Q)}{FE_{NO_2^-}(Q) + FE_{NH_4^+}(Q)} \times 100\% \qquad (3)$$

assuming that NO_2^- and NH_4^+ make up the vast majority of the reaction products.

UHV XPS Measurements

Two foils (Goodfellow), one composed of 64% iron and 36% nickel, and the other composed of 20% iron and 80% nickel by weight, were used as cathodes in a CA experiment designed to mimic the conditions of the conversion measurements described above, with the goal of performing an ex situ analysis of their surface compositions using angle-resolved x-ray photoelectron spectroscopy (XPS). To prepare the foils for usage as cathodes, silver paste was used to electrically contact the foils to sections of wire with the same nickel and iron composition, and epoxy was used to coat the joint area and most of the wire, so that only the foil surface makes electrical contact with the electrolyte.

The foil cathodes were cleaned by abrasion with fine-grit sandpaper and rinsed with 18.2 MΩ water. Then, a two-beaker H-cell was assembled using the same materials and electrolyte that was described in the conversion measurement procedure, except with the foil cathodes instead of wire cathodes. With 100 mM of $NaNO_3$ in the electrolyte, the cells were subjected to an applied potential of -0.5 V vs RHE for one hour followed by a 18.2 MΩ water rinse and stored for later analysis.

XPS measurements were carried out using PHI 5600 XPS spectrometer using Al Kα X-ray radiation (1486.6 eV) for excitation. Measurements were conducted with the sample angled first so that photoelectrons were emitted normal to the sample surface, and then with a 12 degree (grazing) take-off angle.

Ambient pressure XPS measurements

Samples were electrodeposited $NiFeO_x$ on Ni foil, noted by the stoichiometry of 0.1 M $NaNO_3$ + 0.01 M $(Fe_{1-x}Ni_x)(NO_3)_{3-x}$ electrodeposition bath composition. Ambient pressure XPS

measurements were performed at the Advanced Lightsource beamline 9.3.2.[4] Samples were mounted to a ceramic heater button and electronically grounded by an Ir thermocouple. The main spectrometry chamber had a base pressure around 6 x 10^{-10} Torr. Samples were cleaned in 1 x 10^{-6} Torr O_2 at 350 °C while monitoring C 1*s* until featureless. Samples were then cooled to room temperature and characterized under ultrahigh vacuum conditions and in the presence of 1 mTorr nitric oxide (NO, Praxair, 99.5%). Spectra were collected with a 100 eV analyzer pass energy at 0.2 s/step and 100 meV step size. NO and O_2 were dosed into the main chamber via a leak valve.

DFT

Periodic density functional theory (DFT) calculations were carried out with Vienna Ab Initio Simulation Package (VASP).[5–8] The Kohn-Sham equations are solved self-consistently with electron exchange and correlation described by Perdew, Burke, and Ernzerhof functional (PBE) with the Projector Augmented Wave (PAW) method.[9,10] The calculated bulk lattice constants for fcc Ni and bcc Fe were 2.83 and 3.51 Å, respectively, which are similar to tabulated experimental values (2.87 Å and 3.52 Å).[11] The lowest energy surface was modeled for each metal: Ni (111), Fe (110). A five-atomic-layer slab with (3 X 3 X 5) unit cell was used to represent each metal surface. The adsorbates and the three topmost layers were allowed to relax, while the bottom two layers were constrained in their bulk arrangement. A plane wave energy cutoff of 520 eV was used with a 3 X 3 X 1 k-point set for all surfaces. This k-point mesh was chosen by performing a series of test calculations for the energy of an Fe(110) slab using N x N x 1 k-point mesh, starting with N = 1 and incrementing it until the difference in calculated energies from successive runs was less than 0.1 eV.

Periodic slabs are separated in the z direction with a vacuum layer of at least 12 Å to minimize spurious interactions between periodic images. Spin polarization and Methfessel-Paxton scheme[12] with energy smearing of 0.2 eV were applied for all calculations.

Adsorption energies (E_{ads}) for NO_3*, NO_2 *, NO*, N*, O*, H*, N_2*, and NH* were calculated as

$$E_{ads} = E_{ads+slab} - E_{slab} - E_{ads_g} \quad (4)$$

where $E_{ads+slab}$ is the total energy of the slab with the adsorbate, E_{slab} is the total energy of clean metal slab, and $E_{ads,g}$ is the total energy of most stable configuration of adsorbate in gas phase. Free energies of adsorption were determined from DFT-calculated adsorption energies corrected with zero-point energy (ZPE) and standard state entropy corrections (T*S) using the harmonic oscillator approximation at 293K.

Substitution energies for the surface alloy slabs (E_{sub}) were calculated as:

$$E_{sub} = E_{SA} - E_{HS} - E_{DG} + E_{HG} \quad (5)$$

where E_{SA} is the total energy of the surface alloyed slab, E_{HS} is the energy of the host slab, E_{DG} is the energy of the dopant atoms in the gas phase, and E_{HG} is the energy of the substituted host metal atoms in the gas phase.

Activation barriers for nitrite dissociation and nitric oxide dissociation on metal slabs were calculated using the climbing image nudged elastic band (CI-NEB) method[13,14], with six moving images between the initial and final states. The convergence criteria for the adsorption energy and CI-NEB calculations were a maximum residual force of 0.04 eV/Å and 0.06 eV/Å, respectively. In some cases, dimer method[15] calculations were performed starting with nearly converged saddle point images from CI-NEB calculations to help convergence to the transition state. Vibrational modes were calculated for each initial, final, and transition state. Configurations with one imaginary mode were confirmed as transition states, and those with none were confirmed as stable initial or final states.

Surface slab d-band centers were extracted from density of states (DOS) calculations performed using an 8x8x1 k-point mesh. INCAR files containing specific calculation parameters are available upon request.

Table S3: Adsorption energies (E_{ads}) in eV and preferred sites for various adsorbates. All adsorption energies were calculated using equation 4. Abbreviations indicate the preferred adsorption site: face-centered cubic hollow (fcc), long bridge (lb), hollow, bidentate (b; dentate atoms indicated), and tridentate (t; dentate atoms indicated). Only configurations in which the adsorbates are directly bonded to each dopant atom are reported.

Adsorbate	Ni(111)	s-Fe/Ni	d-Fe/Ni	Fe(110)	s-Ni/Fe	d-Ni/Fe
H	-0.56 *fcc*	-0.51 *fcc*	-0.49 *fcc*	-0.65 *lb*	-0.60 *hollow*	-0.54 *hollow*
N	0.82 *fcc*	0.89 *fcc*	0.76 *fcc*	-0.28 *top; hollow*	0.07 *hollow*	0.27 *hollow*
O	-2.87 *fcc*	-2.88 *fcc*	-3.10 *fcc*	-3.89 *hollow*	-3.50 *hollow*	-3.21 *hollow*
NO	-1.04 *fcc*	-0.90 *fcc*	-0.89 *fcc*	-1.37 *hollow*	-1.10 *hollow*	-0.94 *hollow*
NO_2	-1.77 *N,O-b*	-1.84 *N,O-b*	-1.95 *O,O-b*	-2.57 *N,O,O-t*	-2.27 *N,O,O-t*	-2.02 N,O,O-*t*
NO_3	-2.96 *O,O-b*	-3.06 *O,O-b*	-3.22 *O,O-b*	-3.46 *O,O-b*	-3.26 *O,O-b*	-3.09 *O,O-b*
NH	0.03 *fcc*	0.04 *fcc*		-0.75 *lb*	-0.47 *lb*	
N_2	1.71 *top*	1.89 *Fe top*		1.54 *top*	1.78 *Ni top*	

Preferred adsorption sites and trends in adsorption energies for undoped Ni(111) and Fe(110) are consistent with values from similar calculations in the literature.[16–18]

Discussion of mixed site modeling:

To better understand the role of neighboring Ni and Fe sites on adsorption, we performed DFT calculations on simplified surface slabs representing different compositions of the threefold hollow sites, where most relevant adsorbates adsorb (**Table S3**). The different adsorptions sites are comprised of single (s-) or double (d-) atoms of the noted element in the host matrix. For example, d-Fe/Ni represents a hollow site made up of two adjacent Fe atoms and one Ni atom on a Ni(111) surface. Our focus on local site composition is motivated by the fact that NO_3RR intermediates and reactants preferentially adsorb at hollow sites (H*, N*, NO*, NH*) or in bi- or tridentate configurations (NO_3*, NO_2*), where they interact with multiple adjacent surface atoms.

Angle-resolved XPS measurements confirm that the surface composition closely matches the bulk stoichiometry, with a slight Fe enrichment. This rules out scenarios where the surface is dominated by either Ni or Fe. However, XPS does not provide spatial resolution at the atomic scale and cannot distinguish between dispersed atomic configurations and clustering into elemental islands.

To assess how Fe and Ni atoms are likely distributed on alloy surfaces, we calculated the energy required to substitute surface atoms in Ni(111) and Fe(110) – the most thermodynamically stable facets for each metal – with atoms of the other element. Substitution is energetically favorable at low (<50%) concentrations, particularly when dopant atoms are non-adjacent, indicating a thermodynamic preference for dispersion over clustering. Because each surface atom contributes to multiple neighboring threefold hollow sites, surfaces of alloys with roughly balanced Ni and Fe compositions likely have more mixed-metal (2Fe1Ni or 2Ni1Fe) hollow sites than pure-metal (3Ni or 3Fe) sites.

To quantify this expectation, we simulated randomly distributed NiFe surfaces on a close-packed hexagonal lattice across a range of compositions (Fe atom fractions 0, 0.1, 0.2 ... 0.9, 1.0) and counted the occurrences of each threefold hollow site motif. For each composition, 50 different 20×20 atom arrangements were generated and averaged. The resulting distribution of site motifs as a function of overall composition is shown in **Figure S3**. At dilute compositions, three-atom sites of the majority element dominate. However, increasing the minority element concentration to approximately 20% produces a crossover where mixed sites become more abundant than single-element sites, reflecting the statistical tendency towards surface dispersion. Notably, these simulated arrangements treat atom placement as random and do not incorporate the substitution energetics calculated above and shown in **Table S4**, which favor dispersion. Accounting for these energetics would further increase the prevalence of mixed sites and decrease single-element sites at each composition, particularly in the intermediate

composition range relevant to our experimental alloys.

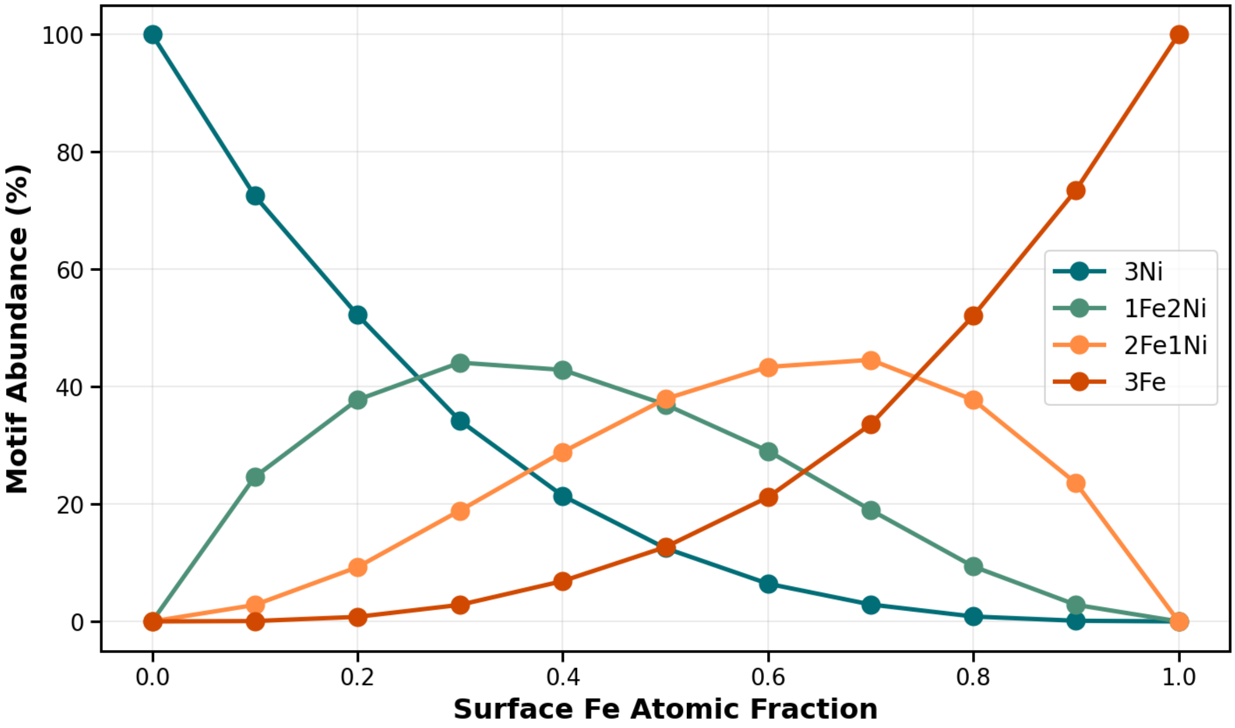

Figure S3: Relative abundance of 3Ni, 1Fe2Ni, 2Fe1Ni, and 3Fe hollow sites on a simulated close-packed hexagonal surface consisting of randomly distributed Ni and Fe atoms, as a function of overall surface composition. Decreasing the concentration of the majority element to below approximately 0.8 results in mixed motifs outnumbering pure majority-element motifs.

Our DFT calculations compare adsorption of key species across the distinct threefold hollow site types on both Ni(111) and Fe(110) host lattices, capturing the full range of active sites likely to occur on real NiFe alloy surfaces. This framework allows us to isolate the effects of local coordination as well as host lattice influences on adsorbate binding and reaction energetics relevant to nitrate reduction.

Table S4: Surface Slabs and Substitution Energies (E_{sub}) for Fe in Ni(111) and Ni in Fe(111). E_{sub} values were calculated using equation 5.

**Ni(111) slab**		**E_{sub} per Fe (eV)**	**Fe(110) slab**		**E_{sub} per Ni (eV)**
No Fe	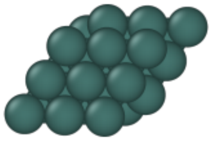	–	No Ni	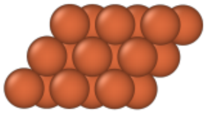	–
with 1 Fe	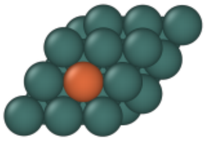	-0.46	with 1 Ni	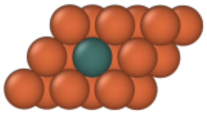	-0.29
with 2 Fe (clustered)	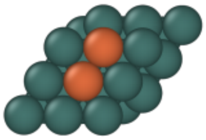	-0.32	with 2 Ni (clustered)	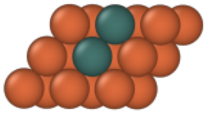	-0.25
with 2 Fe (dispersed)	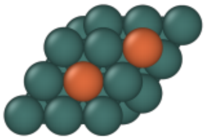	-0.36	with 2 Ni (dispersed)	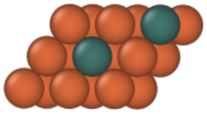	-0.25
with 3 Fe (clustered)	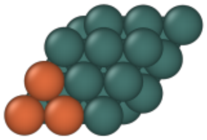	-0.21	with 3 Ni (clustered)	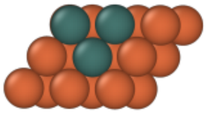	-0.24
with 3 Fe (dispersed)	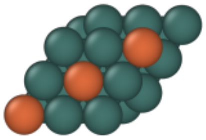	-0.30	with 3 Ni (dispersed)	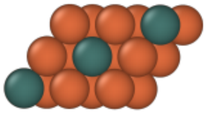	-0.21
with Fe monolayer	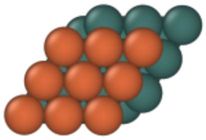	0.07	with Ni monolayer	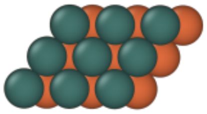	-0.12

Relative Nitrate Adsorption definition:

The "relative NO_3* adsorption" ($\Delta E_{ads,i\ (H\text{-}NO3)}$) conveys the relative adsorption energies of H* and NO_3* on a surface *i* relative to H + NO_3 adsorption on Ni(111) and is defined as:

$$\Delta E_{ads,i\ (H-\ NO_3)} = \Delta E^{*}_{ads,i\ (H-\ NO_3)} - \Delta E^{*}_{ads,i\ (H-\ NO_3),Ni(111)} \tag{7}$$

where

$$\Delta E^{*}_{ads,i\ (H-\ NO_3)} = \Delta E_{ads,i\ (H)} - \Delta E_{ads,i\ (NO3)} \tag{8}$$

with the ΔE_{ads} terms on the right hand side of equation 8 determined using equation 4.

Relative nitrate adsorption is defined with Ni(111) as the reference value, so that a positive value indicates that an adsorption site binds NO_3* more strongly, and/or H* more weakly, than Ni(111) does. However, since the adsorption energy of H* varies only slightly (~0.1 eV) between the surface sites considered herein, the differences in $\Delta E_{ads,i\ (H\ -\ NO3)}$ are mainly due to differences in the adsorption energy of NO_3*.

Assessing possible solvation effects:

To assess the influence of the aqueous environment, we performed additional calculations incorporating implicit solvation using a nonlinear Poisson-Boltzmann continuum model as implemented in VASPsol++.[19] Solvation systematically weakens NO_3* binding by ~0.12-0.14 eV across all motifs while leaving H* adsorption energies essentially unchanged (**Figure S4**). Importantly, this systematic correction does not alter the relative trends in adsorption energies or activation barriers across different surface motifs.

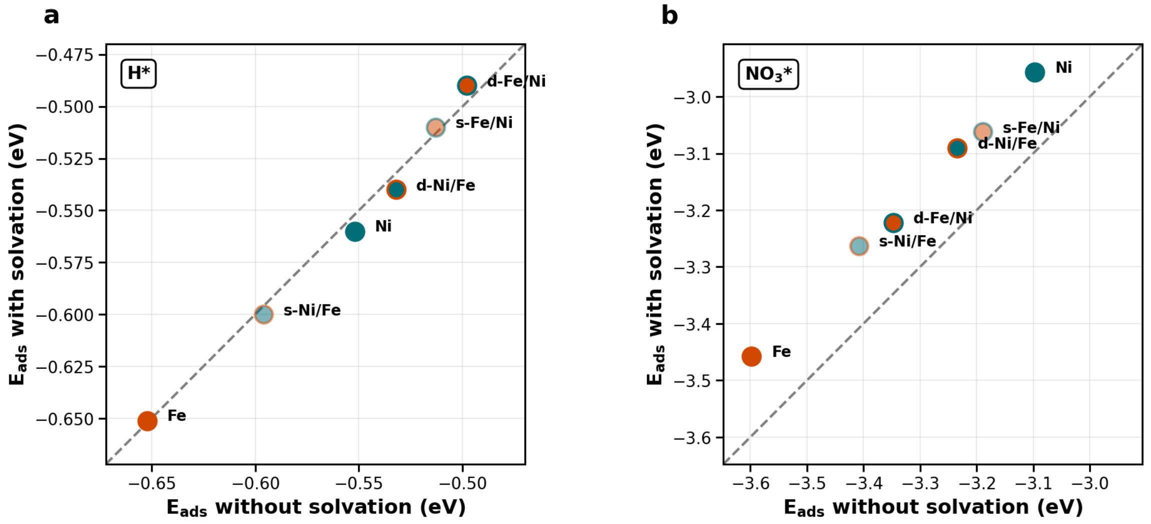

Figure S4: Comparisons of a) H* and b) NO_3* adsorption energies calculated with and without implicit solvation.

NO_3RR rate order with respect to NO_3^- concentration

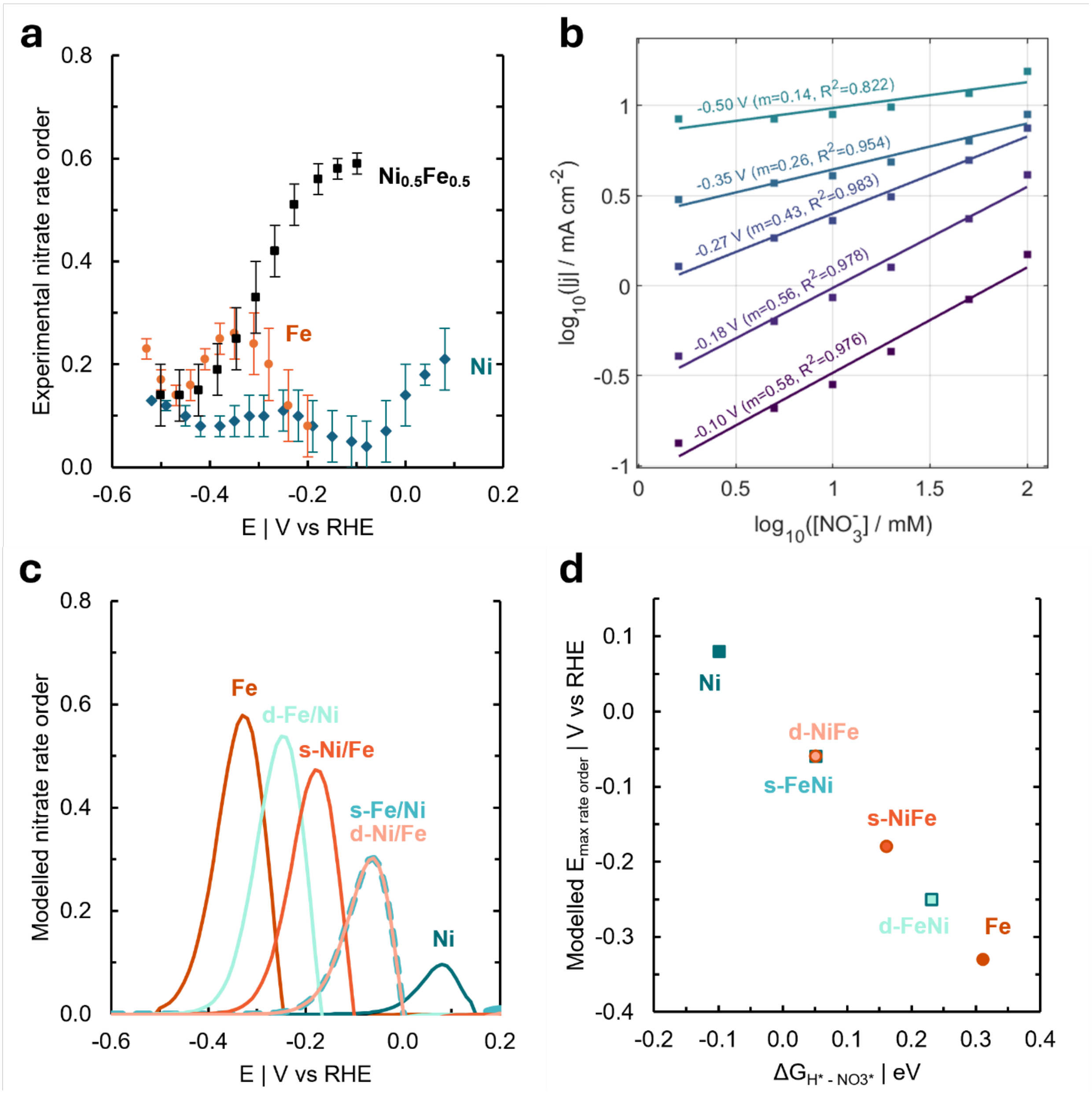

Figure S5: a) Experimentally determined NO_3RR rate order with respect to NO_3^- concentration across a range of potentials for $Ni_{0.5}Fe_{0.5}$ (measured here), Ni, and Fe (adapted from Carvalho et. al[1] with permission. Copyright 2022 American Chemical Society); b) logarithmic graph of steady state current density vs nitrate concentration measured for $Ni_{0.5}Fe_{0.5}$ at five representative potentials – slope of linear fit used to determine rate orders in panel a; c) Modeled NO_3RR rate order (with respect to NO_3^- concentration) versus electrochemical potential, using parameters listed in **Table S4**; (d) Relation between the modeled potential of maximum rate order, $E_{max\ rate\ order}$, and the relative nitrate adsorption energy, $\Delta G_{H^*\text{-}NO3^*}$.

To model the reaction rate order, we leverage our previous microkinetic model[1] that considers competitive adsorption of nitrate and protons, as well as their reduction (modeling partial reduction to nitrite). In our previous work[1] we established that kinetic parameters impact the *magnitude* of the reaction rate order, but only thermodynamic parameters influence the *potential of the maximum* in the reaction rate order. Our conclusions drawn from modeling in this work are based only on the potential dependence of the maximum in rate order and not on interpreting the absolute value of the reaction rate order; as such, we hold kinetic parameters constant for simplicity. Adsorption free energy and kinetic parameters for Ni(111) are based on data derived from this prior rate order analyses.[1] The difference between $\Delta G_{H^*-NO_3^*}$ calculated here in vacuum (231.6 kJ/mol) and measured in an aqueous environment (-9.5 kJ/mol)[1] is then applied to the parameters calculated for all other surface geometries and compositions considered here (**Table S5**), giving the modeled rate orders shown in panel c of **Figure S3**. The experimental rate order curve shown in panel a of **Figure S3** (limited to electrochemical potentials below which Fe is expected to remain metallic) is similar to an average of possible site motifs, resulting in a wider range of potentials over which NO_3RR rate orders are above the baseline.

Table S5. Parameters used to produce microkinetic model results in **Figure S4**.

	Metals	Ni(111)	s-FeNi	d-FeNi	s-NiFe	d-NiFe	Fe(110)
Conc. and Selectivity	α_{H^+} | M	0.1	0.1	0.1	0.1	0.1	0.1
	$\alpha_{NO_3^-}$ | M	0.1	0.1	0.1	0.1	0.1	0.1
	n_{HER}	2	2	2	2	2	2
	n_{NO_3RR}	2	2	2	2	2	2
Thermo	K_H	$5.0\text{x}10^{3}$	$6.9\text{x}10^{2}$	$3.1\text{x}10^{2}$	$2.4\text{x}10^{4}$	$2.3\text{x}10^{3}$	$1.8\text{x}10^{5}$
	ΔG_{H*} | kJ/mol	-20.8	-15.9	-14.0	-24.6	-18.8	-29.4
	$K_{NO_3^-}$	$1.0\text{x}10^{2}$	$5.3\text{x}10^{3}$	$3.0\text{x}10^{6}$	$1.4\text{x}10^{7}$	$1.7\text{x}10^{4}$	$4.0\text{x}10^{10}$
	$\Delta G_{NO_3^-*}$ | kJ/mol	-11.2	-20.9	-36.3	-40.2	-23.8	-59.5
	$K_H/K_{NO_3^-}$	$5.0\text{x}10^{1}$	$1.3\text{x}10^{-1}$	$1.1\text{x}10^{-4}$	$1.7\text{x}10^{-3}$	$1.3\text{x}10^{-1}$	$4.5\text{x}10^{-6}$
	$\boldsymbol{\Delta G_{H*} - \Delta G_{NO_3^-*}}$	-9.5	4.9	22.3	15.6	4.9	30.0
Kinetics	η_{HER} | V_{RHE}	0	0	0	0	0	0
	η_{NO_3RR} | V_{RHE}	0.82	0.82	0.82	0.82	0.82	0.82
	α	0.21	0.21	0.21	0.21	0.21	0.21
	k_{1F}^0 | L/s-cm^2	$5.0\text{x}10^{-10}$	$5.0\text{x}10^{-10}$	$5.0\text{x}10^{-10}$	$5.0\text{x}10^{-10}$	$5.0\text{x}10^{-10}$	$5.0\text{x}10^{-10}$
	k_{2F}^0 | L/s-cm^2	$1.0\text{x}10^{-10}$	$1.0\text{x}10^{-10}$	$1.0\text{x}10^{-10}$	$1.0\text{x}10^{-10}$	$1.0\text{x}10^{-10}$	$1.0\text{x}10^{-10}$
	k_{3F}^0 | mol/s-cm^2	$1.0\text{x}10^{-10}$	$1.0\text{x}10^{-10}$	$1.0\text{x}10^{-10}$	$1.0\text{x}10^{-10}$	$1.0\text{x}10^{-10}$	$1.0\text{x}10^{-10}$
	k_{5F}^0 | L^2/mol-s-cm^2	$9.9\text{x}10^{-12}$	$9.9\text{x}10^{-12}$	$9.9\text{x}10^{-12}$	$9.9\text{x}10^{-12}$	$9.9\text{x}10^{-12}$	$9.9\text{x}10^{-12}$
	k_{6F}^0 | mol/s-cm^2	$6.1\text{x}10^{-12}$	$6.1\text{x}10^{-12}$	$6.1\text{x}10^{-12}$	$6.1\text{x}10^{-12}$	$6.1\text{x}10^{-12}$	$6.1\text{x}10^{-12}$
	k_{7F}^0 | L/s-cm^2	$6.1\text{x}10^{-12}$	$6.1\text{x}10^{-12}$	$6.1\text{x}10^{-12}$	$6.1\text{x}10^{-12}$	$6.1\text{x}10^{-12}$	$6.1\text{x}10^{-12}$

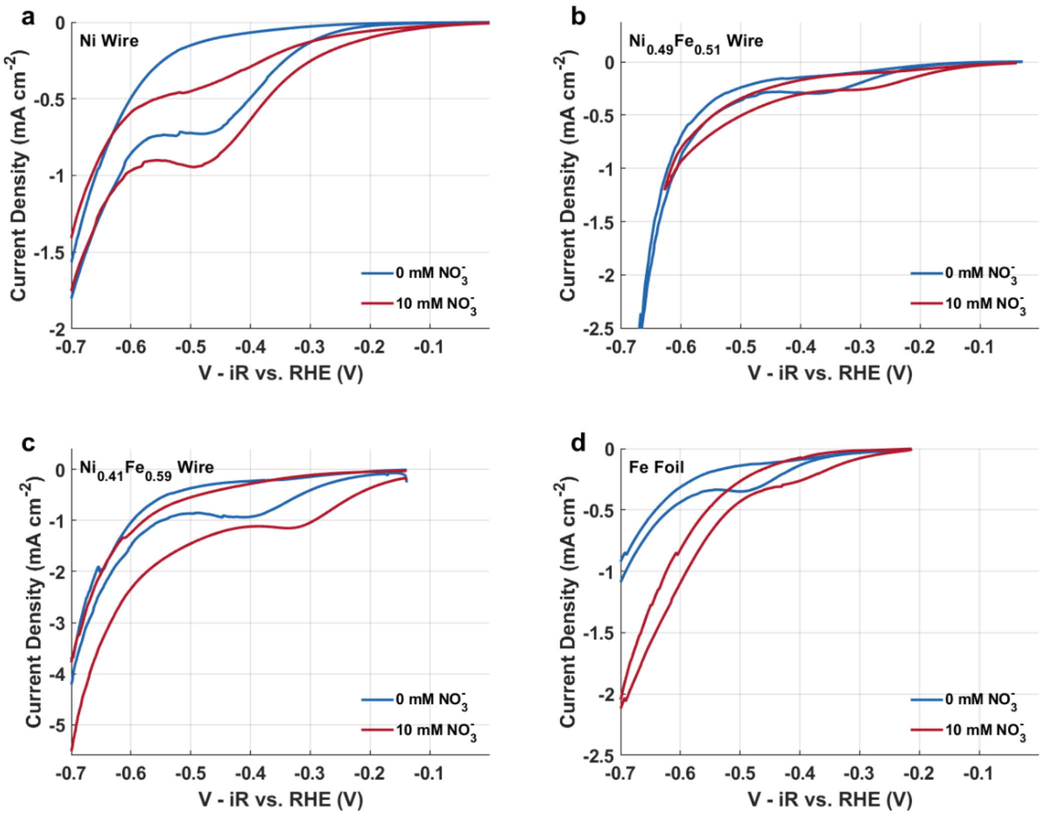

Figure S6: Representative CV sweeps (unstirred) for HER only (blue) and with nitrate (red) of a) Ni wire, b) $Ni_{0.49}Fe_{0.51}$ wire, c) $Ni_{0.41}Fe_{0.59}$ wire, and d) Fe foil in 100 mM pH 7 sodium phosphate buffer with and without 10 mM of sodium nitrate, with a scan rate of 10 mV/s. The mass transfer limited feature around -0.4 V vs RHE arises from phosphate deprotonation.[1] Current densities are normalized by electrochemical surface area as determined from capacitance measurements (**Figure S7**).

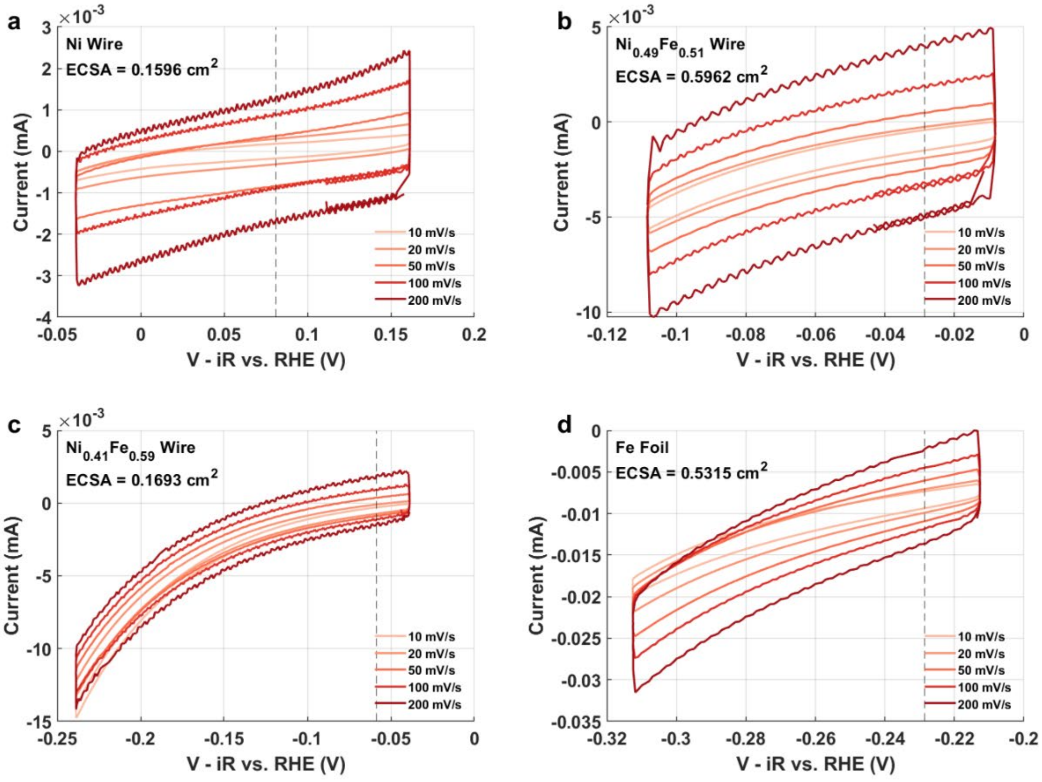

Figure S7. Measurement of double layer capacitance for estimation of ECSA; the current at the potential indicated by the dashed line was used as a function of scan rate to extract the capacitance, accounting for any minor Faradaic baseline giving rise to asymmetry in the cathodic and anodic sweep. The surface area for normalization of CVs was estimated assuming a specific capacitance of 40 μF/cm^2 [20]a) Ni, b) $Ni_{0.49}Fe_{0.51}$, c) $Ni_{0.41}Fe_{0.59}$, d) Fe.

APXPS shows NiFe oxides adsorb more NO_x than FeO_x or NiO_x

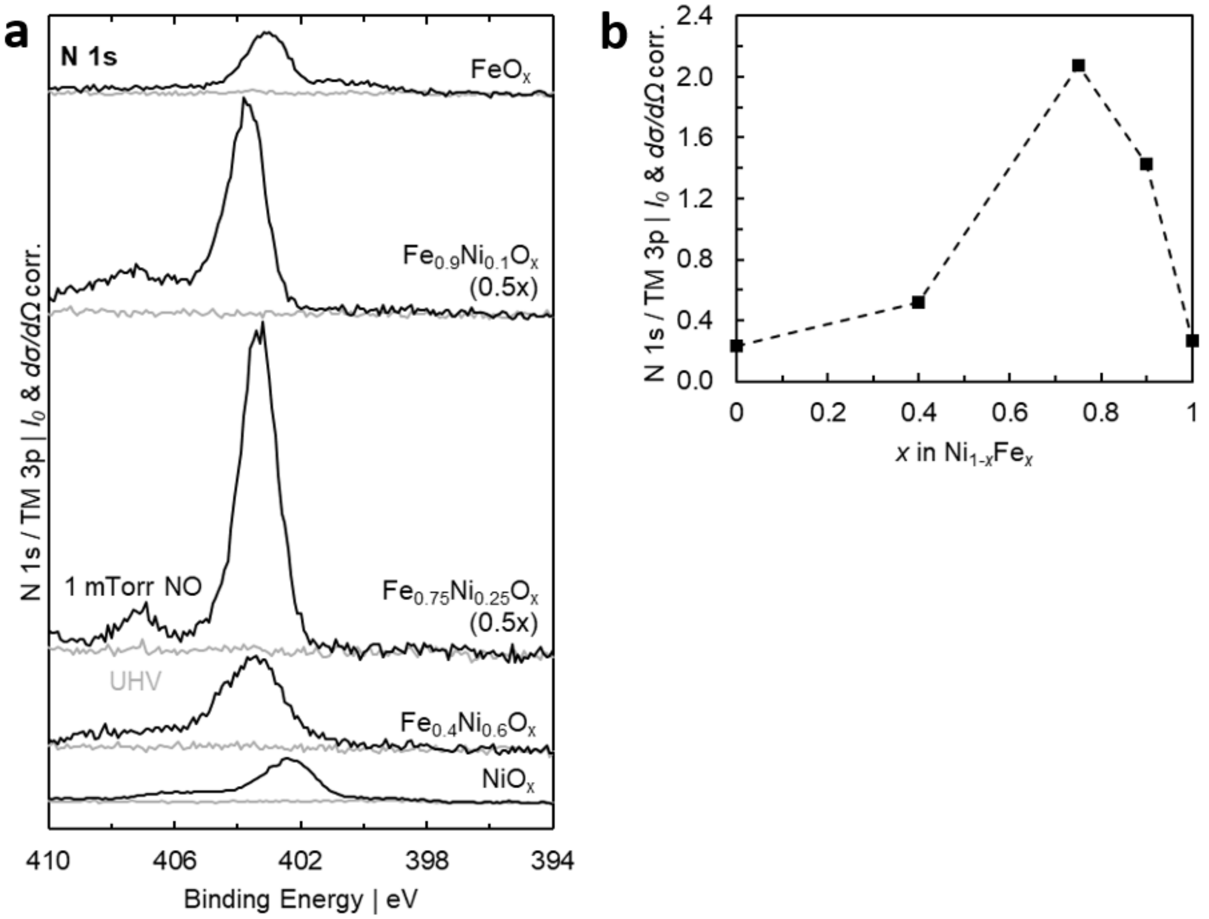

Figure S8: a) Core level N 1s spectra (560 eV *hv*) for a series of electrodeposited Fe_xNi_{1-x} alloy oxides after cleaning in ultrahigh vacuum (UHV) (light gray trace) and during exposure to 1 mTorr of gaseous NO (black trace). b) Ratio of N 1s and TM 3p intensities (with increasing Fe content: 0.23, 0.52, 2.07, 1.43, 0.27) measured during 1 mTorr NO exposure.

Higher intensities of N 1s spectra for NiFe alloy oxide surfaces compared to NiO_X and FeO_X in the presence of 1 mTorr of NO gas suggest that mixed NiFe surfaces better promote the adsorption of NO_X species, particularly for the more Fe-rich alloy compositions. Spectra were collected using ambient pressure x-ray photoelectron spectroscopy (AP-XPS) measurements alongside, and following the same methods as, the measurements made in [21]. However, here the substrates are electrodeposited NiFe alloys that have been fully oxidized, as opposed to pure (i.e. not oxidized) metal foils. In that work, N 1s spectra were deconvoluted into contributions from various adsorbed nitrogen containing species, with oxidized nitrogen adsorbates (NO*,

NO_2*, NO_3*) generally having binding energies > 400 eV and unoxidized nitrogen adsorbates (N*, NH_X*, CN_X*) generally having binding energies < 400 eV. Here, several peaks are evident, but all of them have binding energy > 400 eV, suggesting that the N 1s signal intensity on NiFe oxides is due primarily to adsorbed NO_X.

Surface composition – Angle Resolved XPS

XPS was used to assess the surface composition of the $Ni_{0.8}Fe_{0.2}$ and $Ni_{0.36}Fe_{0.64}$ (wt%) foils after electrochemical measurements of NO_3RR. To probe different sampling depths, spectra were collected at two emission angles: grazing emission (12°, more surface-sensitive), and normal emission (90° to the surface, less surface-sensitive, more bulk-like). As expected, the grazing-angle spectra showed lower overall signal intensity due to the reduced escape depth of photoelectrons.

To quantify the relative amounts of Ni and Fe at different depths, the Fe 3p and Ni 3p peaks were integrated from both sets of spectra. An *estimated iron fraction* was calculated using the following equation:

$$\text{Estimated Fe Fraction} = \frac{I_{\text{Fe 3p}}/S_{\text{Fe 3p}}}{\left(I_{\text{Fe 3p}}/S_{\text{Fe 3p}}\right) + \left(I_{\text{Ni 3p}}/S_{\text{Ni 3p}}\right)} \tag{9}$$

where I is the measured peak area and S is the tabulated relative sensitivity factor for the corresponding orbital. Relative sensitivity factors for Al Kα excitation were taken from the CasaXPS library[22], based on Scofield photoionization cross-sections.[23]

The apparent Fe fraction measured at grazing emission was slightly higher than at normal emission for both samples (**Figure S9**), suggesting modest Fe enrichment at the surface.

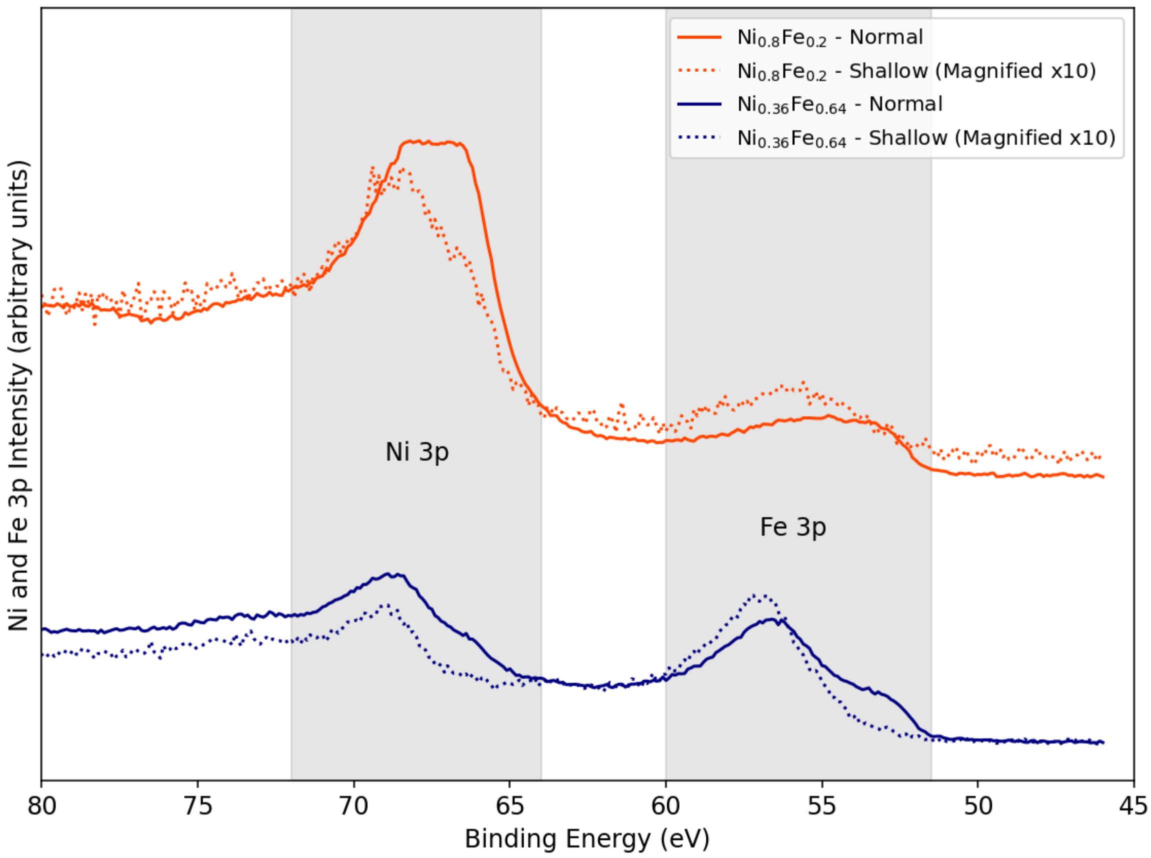

Figure S9: Ni and Fe 3p XPS spectra for $Ni_{0.8}Fe_{0.2}$ and $Ni_{0.36}Fe_{0.64}$ (wt%) foils following NO_3RR measurements measured with normal and shallow (12°) emission. Units for intensity are counts per second (CPS), but in order to aid visual comparison, an offset is applied, and the shallow emission CPS are multiplied by 10 to counteract their weaker photoelectron signal. Although shallow emission (surface sensitive) measurements suggest an oxidized surface (in contrast to a reduced bulk), the ex situ nature of this measurement precludes conclusions regarding the active valence state during electrochemistry.

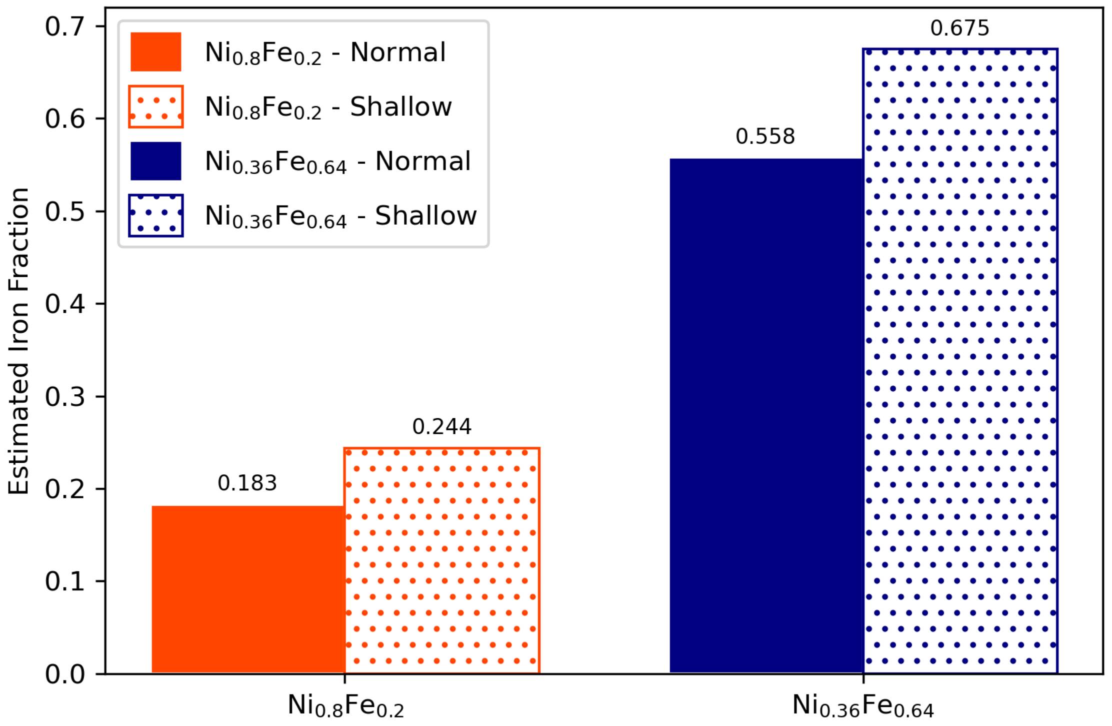

Figure S10: Estimated atomic iron fractions by XPS following NO_3RR measurements of the foil surfaces (from shallow emission) and bulk (from normal emission), where the composition noted on the x-axis and legend is wt%, corresponding to $Ni_{0.79}Fe_{0.21}$ and $Ni_{0.37}Fe_{0.63}$ atomic fraction based on the purchased bulk composition.

Sensitivity of adsorbates to host vs site composition

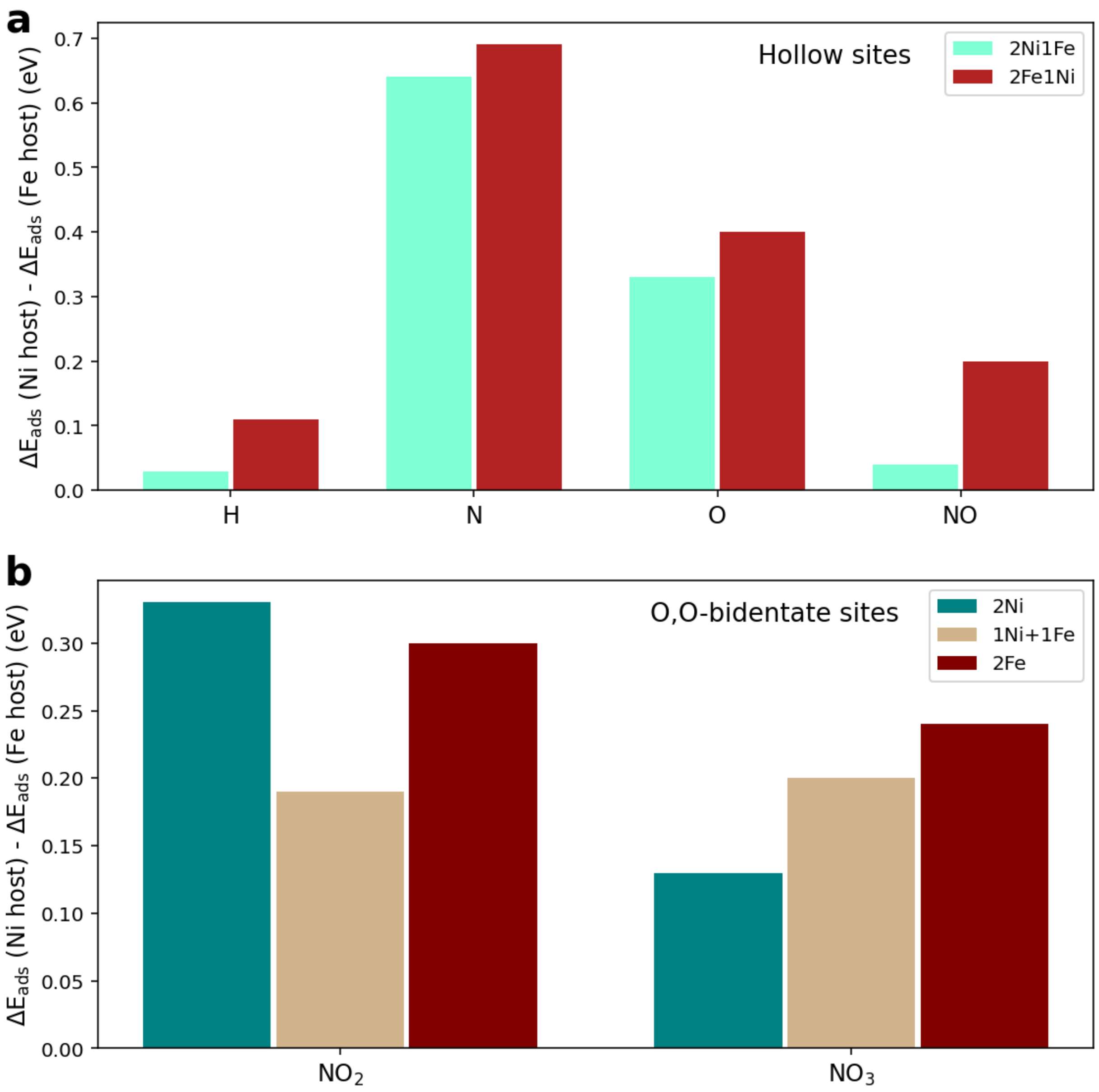

Figure S11: Differences in ΔE_{ads} for various adsorbates on equivalent a) hollow and b) bidentate adsorption sites, on different host lattices (Ni(111) or Fe(110)). All adsorbates bound more strongly (lower ΔE_{ads}) to sites on the Fe(110) lattice. Values shown are calculated as $\Delta E_{ads,\ Ni(111)\ host} - \Delta E_{ads,\ Fe(110)\ host}$.

Shift in d-band energy from host lattice vs surface alloy motif

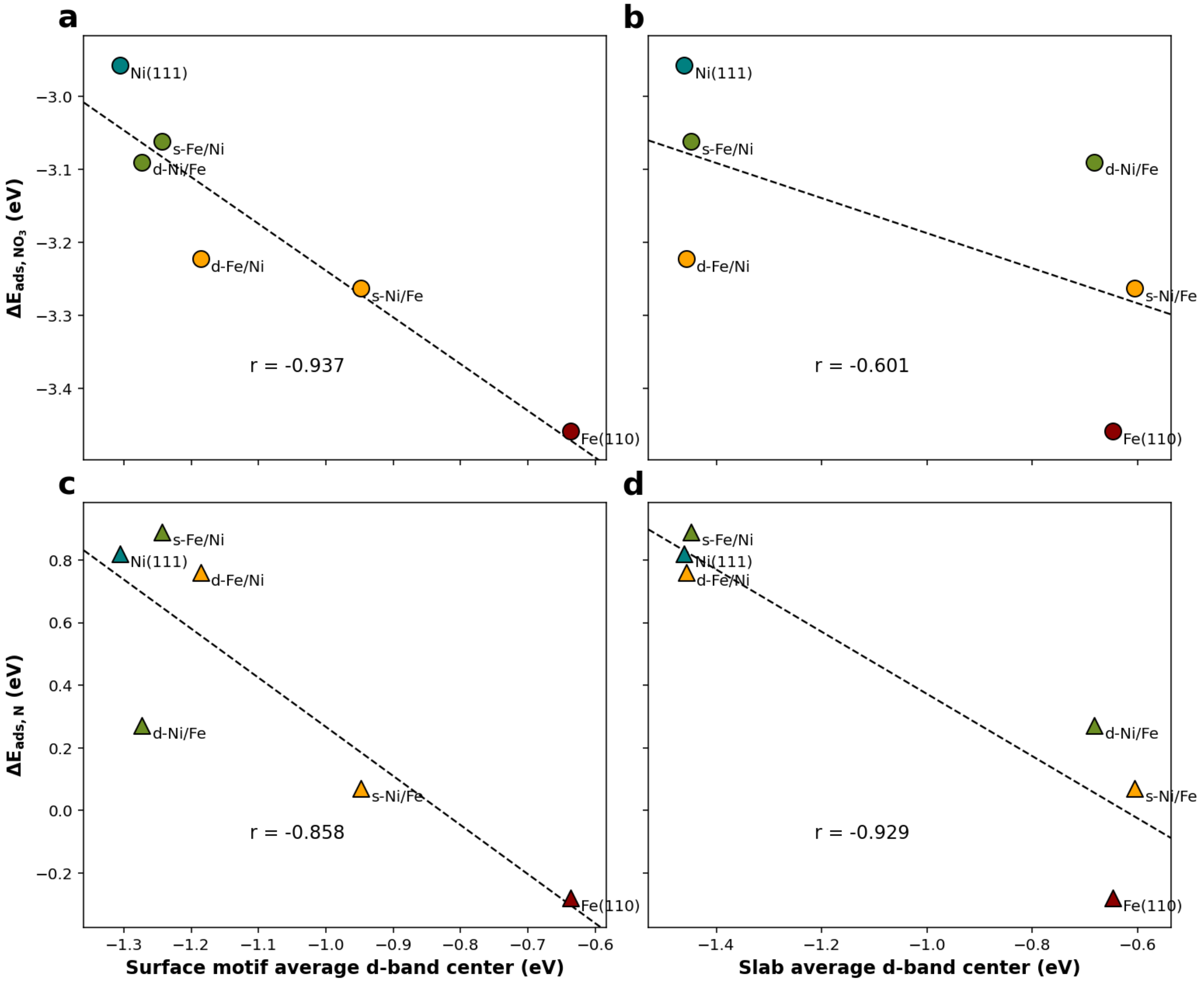

Figure S12: ΔE_{ads} of NO_3 (a,b) and N (c,d) on each surface motif plotted against d-band centers calculated for the entire surface slab (b,d) or averaged from the three surface motif atoms only (a,c). Data points are color coded based on the atom content of the surface motif (3 Fe – red, 2 Fe and 1 Ni – yellow, 2 Ni and 1 Fe – green, 3 Ni – blue). Pearson correlation coefficients (r) are show the linear correlation between these values. There are strong negative correlations (indicated by r values close to -1) between surface motif averaged d-band center and $\Delta E_{ads,NO3}$ (r = -0.937) and between slab average d-band center and $\Delta E_{ads,N}$ (r = -0.929), reflecting the two adsorbates' greater sensitivity to surface and bulk composition, respectively.

NO_2* Deoxygenation and NO* Dissociation BEP Scaling

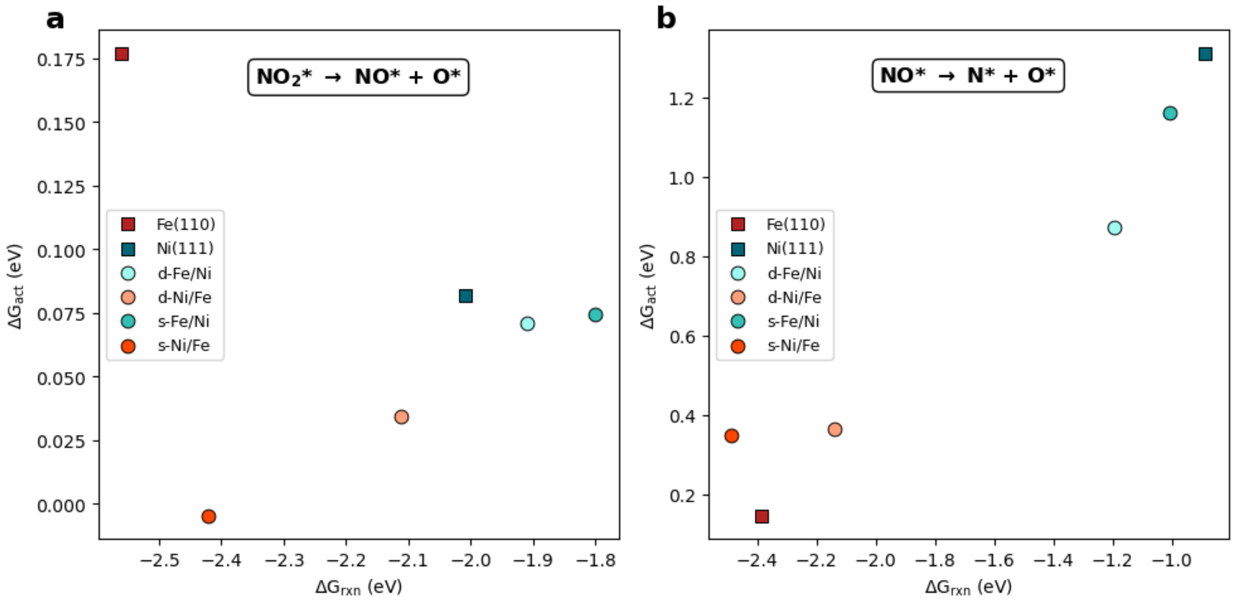

Figure S13: Activation free energy barrier vs free energy of reaction for (a) NO_2* deoxygenation and (b) NO* dissociation steps on each surface motif. For NO* dissociation (b), there is a linear correlation between these values, in line with BEP scaling relations. However, for NO_2* deoxygenation (a), a linear trend is not apparent, suggesting that BEP scaling should not be assumed.

N* Hydrogenation Energy

In addition to the NO_2* deoxygenation and NO* dissociation steps discussed in the main text, we also calculated reaction and activation free energies associated with hydrogenation of an adsorbed nitrogen atom. Both of these values are lower for Ni(111) than for Fe(110) and are lowered significantly when Ni is substituted into the Fe(110) surface. The activation barriers here are quite high. In this model the hydrogen was assumed to adsorb at a hollow site next to the N* hollow site. For the s-Ni/Fe and s-Fe/Ni slabs, N* and H* were both adsorbed at hollow sites adjacent to the substituting atom. Other mechanisms have been proposed[1] in which the proton comes directly from the electrolyte, which would likely result in a different transition state, however, the NH* adsorption energy would be unaffected. The adsorption energy of H* is similar for these surfaces so the relative reaction energies would be similar for the different mechanism.

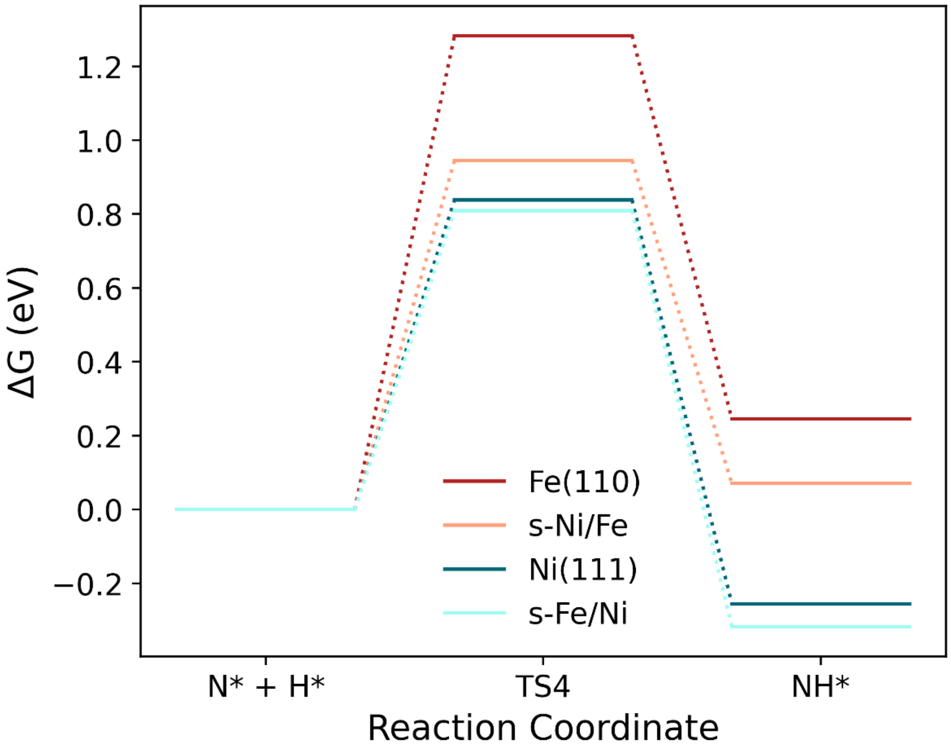

Figure S14: Reaction free energy diagram for N* + H* → NH* on Ni(111), s-Ni/Fe, Fe(111), and s-Fe/Ni

References

(1) Carvalho, O. Q.; Marks, R.; Nguyen, H. K. K.; Vitale-Sullivan, M. E.; Martinez, S. C.; Árnadóttir, L.; Stoerzinger, K. A. Role of Electronic Structure on Nitrate Reduction to Ammonium: A Periodic Journey. *J. Am. Chem. Soc.* **2022**, *144* (32), 14809–14818. https://doi.org/10.1021/jacs.2c05673.

(2) Chen, J.; Zhang, Z.; Hershkovitz, E.; Poplawsky, J.; Dandu, R. S. B.; Hung, C.-Y.; Wang, W.; Yao, Y.; Li, L.; Xin, H.; Kim, H.; Cai, W. Selective Oxidation and Nickel Enrichment Hinders the Repassivation Kinetics of Multi-Principal Element Alloy Surfaces. *Acta Mater.* **2024**, *263*, 119490. https://doi.org/10.1016/j.actamat.2023.119490.

(3) Kang, Q.; Lai, D.; Tang, W.; Lu, Q.; Gao, F. Intrinsic Activity Modulation and Structural Design of NiFe Alloy Catalysts for an Efficient Oxygen Evolution Reaction. *Chem. Sci.* **2021**, *12* (11), 3818–3835. https://doi.org/10.1039/D0SC06716D.

(4) Grass, M. E.; Karlsson, P. G.; Aksoy, F.; Lundqvist, M.; Wannberg, B.; Mun, B. S.; Hussain, Z.; Liu, Z. New Ambient Pressure Photoemission Endstation at Advanced Light Source Beamline 9.3.2. *Rev. Sci. Instrum.* **2010**, *81* (5), 053106. https://doi.org/10.1063/1.3427218.

(5) Kresse, G.; Hafner, J. *Ab Initio* Molecular Dynamics for Liquid Metals. *Phys. Rev. B* **1993**, *47* (1), 558–561. https://doi.org/10.1103/PhysRevB.47.558.
(6) Kresse, G.; Hafner, J. *Ab Initio* Molecular-Dynamics Simulation of the Liquid-Metal–Amorphous-Semiconductor Transition in Germanium. *Phys. Rev. B* **1994**, *49* (20), 14251–14269. https://doi.org/10.1103/PhysRevB.49.14251.
(7) Kresse, G.; Furthmüller, J. Efficient Iterative Schemes for *Ab Initio* Total-Energy Calculations Using a Plane-Wave Basis Set. *Phys. Rev. B* **1996**, *54* (16), 11169–11186. https://doi.org/10.1103/PhysRevB.54.11169.
(8) Kresse, G.; Furthmüller, J. Efficiency of Ab-Initio Total Energy Calculations for Metals and Semiconductors Using a Plane-Wave Basis Set. *Comput. Mater. Sci.* **1996**, *6* (1), 15–50. https://doi.org/10.1016/0927-0256(96)00008-0.
(9) Perdew, J. P.; Burke, K.; Ernzerhof, M. Generalized Gradient Approximation Made Simple [Phys. Rev. Lett. 77, 3865 (1996)]. *Phys. Rev. Lett.* **1997**, *78* (7), 1396–1396. https://doi.org/10.1103/PhysRevLett.78.1396.
(10) Perdew, J. P.; Burke, K.; Ernzerhof, M. Generalized Gradient Approximation Made Simple. *Phys. Rev. Lett.* **1996**, *77* (18), 3865–3868. https://doi.org/10.1103/PhysRevLett.77.3865.
(11) *CRC Handbook of Chemistry and Physics*, 102nd edition 2021-2022.; Rumble, J. R., Ed.; CRC Press: Boca Raton London New York, 2021.
(12) Methfessel, M.; Paxton, A. T. High-Precision Sampling for Brillouin-Zone Integration in Metals. *Phys. Rev. B* **1989**, *40* (6), 3616–3621. https://doi.org/10.1103/PhysRevB.40.3616.
(13) Henkelman, G.; Uberuaga, B. P.; Jónsson, H. A Climbing Image Nudged Elastic Band Method for Finding Saddle Points and Minimum Energy Paths. *J. Chem. Phys.* **2000**, *113* (22), 9901–9904. https://doi.org/10.1063/1.1329672.
(14) Henkelman, G.; Jónsson, H. Improved Tangent Estimate in the Nudged Elastic Band Method for Finding Minimum Energy Paths and Saddle Points. *J. Chem. Phys.* **2000**, *113* (22), 9978–9985. https://doi.org/10.1063/1.1323224.
(15) Henkelman, G.; Jónsson, H. A Dimer Method for Finding Saddle Points on High Dimensional Potential Surfaces Using Only First Derivatives. *J. Chem. Phys.* **1999**, *111* (15), 7010–7022. https://doi.org/10.1063/1.480097.
(16) Bai, Y.; Kirvassilis, D.; Xu, L.; Mavrikakis, M. Atomic and Molecular Adsorption on Ni(111). *Surf. Sci.* **2019**, *679*, 240–253. https://doi.org/10.1016/j.susc.2018.08.004.
(17) Xu, L.; Kirvassilis, D.; Bai, Y.; Mavrikakis, M. Atomic and Molecular Adsorption on Fe(110). *Surf. Sci.* **2018**, *667*, 54–65. https://doi.org/10.1016/j.susc.2017.09.002.
(18) Chen, H.-L.; Wu, S.-Y.; Chen, H.-T.; Chang, J.-G.; Ju, S.-P.; Tsai, C.; Hsu, L.-C. Theoretical Study on Adsorption and Dissociation of NO_2 Molecule on Fe(111) Surface. *Langmuir* **2010**, *26* (10), 7157–7164. https://doi.org/10.1021/la904233b.
(19) Islam, S. M. R.; Khezeli, F.; Ringe, S.; Plaisance, C. An Implicit Electrolyte Model for Plane Wave Density Functional Theory Exhibiting Nonlinear Response and a Nonlocal Cavity Definition. *J. Chem. Phys.* **2023**, *159* (23), 234117. https://doi.org/10.1063/5.0176308.
(20) McCrory, C. C. L.; Jung, S.; Peters, J. C.; Jaramillo, T. F. Benchmarking Heterogeneous Electrocatalysts for the Oxygen Evolution Reaction. *J. Am. Chem. Soc.* **2013**, *135* (45), 16977–16987. https://doi.org/10.1021/ja407115p.
(21) Carvalho, O. Q.; Nguyen, H. K. K.; Padavala, S. K. M.; Árnadóttir, L.; Crumlin, E. J.; Stoerzinger, K. A. Interaction of Nitric Oxide with Late 3d Transition Metals: Dissociation

and Metal Oxidation. *J. Phys. Chem. C* **2024**, *128* (42), 18006–18017. https://doi.org/10.1021/acs.jpcc.4c03048.
(22) Fairley, N.; Fernandez, V.; Richard-Plouet, M.; Guillot-Deudon, C.; Walton, J.; Smith, E.; Flahaut, D.; Greiner, M.; Biesinger, M.; Tougaard, S.; Morgan, D.; Baltrusaitis, J. Systematic and Collaborative Approach to Problem Solving Using X-Ray Photoelectron Spectroscopy. *Appl. Surf. Sci. Adv.* **2021**, *5*, 100112. https://doi.org/10.1016/j.apsadv.2021.100112.
(23) Scofield, J. H. Hartree-Slater Subshell Photoionization Cross-Sections at 1254 and 1487 EV. *J. Electron Spectrosc. Relat. Phenom.* **1976**, *8* (2), 129–137. https://doi.org/10.1016/0368-2048(76)80015-1.
